# Epidemiology of sepsis-associated acute kidney injury in critically ill patients: a multicenter, prospective, observational cohort study in South Korea

**DOI:** 10.1186/s13054-024-05167-9

**Published:** 2024-11-24

**Authors:** Myung Jin Song, Yeonhoon Jang, Matthieu Legrand, Sunghoon Park, RyoungEun Ko, Gee Young Suh, Dong Kyu Oh, Su Yeon Lee, Mi Hyeon Park, Chae-Man Lim, Se Young Jung, Sung Yoon Lim, The Korean Sepsis Alliance (KSA) investigator

**Affiliations:** 1 Department of Internal Medicine, Seoul National University College of Medicine and Seoul National University Bundang Hospital, 82 Gumi-ro, Bundang-gu, Seongnam-si , 13620 Republic of Korea; 2 Office of Hospital Information, Seoul National University Bundang Hospital, Seongnam-si, Republic of Korea; 3 Department of Anesthesia and Perioperative Care, Division of Critical Care Medicine, University of California, San Francisco, CA USA; 4 Department of Pulmonary, Allergy and Critical Care Medicine, Hallym University Sacred Heart Hospital, Anyang, Republic of Korea; 5 Department of Critical Care Medicine, Samsung Medical Center, Sungkyunkwan University School of Medicine, Seoul, Republic of Korea; 6 Division of Pulmonary and Critical Care Medicine, Department of Medicine, Samsung Medical Center, Sungkyunkwan University, Seoul, Republic of Korea; 7 Division of Pulmonology and Critical Care Medicine, Department of Internal Medicine, Asan Medical Center, University of Ulsan College of Medicine, Seoul, Republic of Korea; 8 Department of Family Medicine, College of Medicine, Seoul National University, Seoul, Republic of Korea

**Keywords:** Acute kidney injury, Epidemiology, Intensive care unit, Kidney, Sepsis

## Abstract

**Background:**

Despite the clinical importance of sepsis-associated acute kidney injury (SA-AKI), little is known about its epidemiology. We aimed to investigate the incidence and outcomes of SA-AKI, as well as the risk factors for mortality among patients with severe SA-AKI in critically ill patients.

**Methods:**

This secondary multicenter, observational, prospective cohort analysis of sepsis in South Korea evaluated patients aged ≥ 19 years admitted to intensive care units with a diagnosis of sepsis. The primary outcome was the incidence of SA-AKI, defined using the new consensus definition of the Acute Disease Quality Initiative 28 Workgroup. Secondary outcomes were in-hospital mortality and risk factors for in-hospital mortality.

**Results:**

Between September 2019 and December 2022, 5100 patients were admitted to intensive care units with a diagnosis of sepsis, and 3177 (62.3%) developed SA-AKI. A total of 613 (19.3%), 721 (22.7%), and 1843 (58.0%) patients had stage 1, 2, and 3 SA-AKI, respectively. Severe SA-AKI (stages 2 and 3 combined) was associated with an increased risk of in-hospital mortality. Adherence to the fluid resuscitation component of the one-hour sepsis bundle was associated with a decreased risk of in-hospital mortality in severe SA-AKI (adjusted odds ratio, 0.62; 95% confidence interval, 0.48–0.79; *P* < 0.001).

**Conclusions:**

Of the patients admitted to the intensive care unit for sepsis, 62.3% developed SA-AKI. Severe SA-AKI was associated with an increased risk of mortality. Adherence to the fluid resuscitation component of the one-hour sepsis bundle can potentially improve outcomes in these patients.

**Supplementary Information:**

The online version contains supplementary material available at 10.1186/s13054-024-05167-9.

## Background

Sepsis is a life-threatening organ dysfunction caused by a dysregulated host response to infection [1], responsible for 20% of all deaths globally [2]. The kidney is a common target organ for progressive organ dysfunction in sepsis, and approximately 50% of all acute kidney injury (AKI) cases in the intensive care unit (ICU) are triggered by sepsis [3, 4]. AKI caused by sepsis is associated with a worse prognosis than AKI due to any other causes [4–6].

Sepsis induces kidney injury through several mechanisms, including systemic and renal inflammation, metabolic reprogramming, and microcirculatory dysfunction [7, 8]. Kidney injury in patients with sepsis can also result from the indirect mechanisms driven by sepsis treatment and subsequent sequelae [9]. Collectively, AKI occurring in the context of sepsis is referred to as sepsis-associated AKI (SA-AKI).

The epidemiology of SA-AKI is inconsistent owing to the lack of a standard definition. A systematic review of 47 studies reported an incidence ranging from 14 to 87%, with varying definitions for sepsis and AKI [10]. Recently, the Acute Disease Quality Initiative (ADQI) 28 Workgroup introduced a consensus definition of SA-AKI, which stipulates that both sepsis (defined by the Sepsis-3 definition) [1] and AKI (defined by the Kidney Disease: Improving Global Outcomes [KDIGO] guidelines) [11] should be present, with AKI occurring within seven days of the diagnosis of sepsis [8].

To improve the epidemiological understanding of SA-AKI in line with the consensus definition by the ADQI 28 Workgroup, this study aimed to investigate the incidence and outcomes of SA-AKI, as well as the risk factors for mortality among patients with severe SA-AKI using prospective observational data from patients with sepsis that reflects real-world practice.

## Methods

### Study design and cohort

**Fig. 1:**
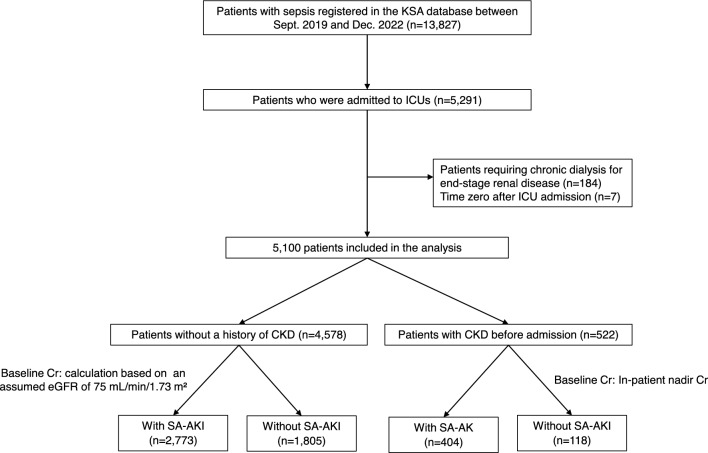
Study flow CKD, chronic kidney disease; ESRD, end-stage renal disease; ICU, intensive care unit; KSA, Korean Sepsis Alliance; SA-AKI, sepsis-associated acute kidney injury; Cr, creatinine level

This study was a secondary analysis of the Korean Sepsis Alliance (KSA) registry, a prospective observational cohort. A detailed explanation of the registry is provided in e-Method 1 [12]. The cohort included patients from 21 tertiary or university-affiliated hospitals in South Korea between September 2019 and December 2022. All consecutive adult patients (age ≥ 19 years) who presented to the emergency room or patients admitted to general wards were screened for eligibility and included in the cohort when they were diagnosed with sepsis. Patients who were admitted to an ICU were selected for analysis. Of note, patients who developed sepsis during an ICU stay were not included in the study (Fig. 1).

### Ethics statements

This study was approved by the institutional review boards of each participating hospital, and the requirement for informed consent was waived because of the noninterventional, observational nature of the study. We followed the Strengthening the Reporting of Observational Studies in Epidemiology (STROBE) guidelines for observational cohort studies [13].

### Outcomes and variables

The primary outcome was the incidence of SA-AKI. Secondary outcomes were in-hospital mortality and risk factors for in-hospital mortality. Further details on the study variables are described in Supplementary e-Method 2 and e-Table 1.

### *Sepsis* and time zero identification

The Sepsis-3 criteria were used for diagnosing sepsis [1]. Patients arriving at the emergency room were screened for possible infections using the quick Sequential Organ Failure Assessment (qSOFA) score. Those with a qSOFA score of ≥ 2 were further evaluated for organ dysfunction using the full SOFA score. For hospitalized patients, those who were managed by a rapid response team and identified as having sepsis were assessed using the full SOFA score. A diagnosis of sepsis was confirmed if the patient satisfied the following two criteria: 1) probable or confirmed diagnosis of infection, and 2) an increase in the SOFA score of ≥ 2. Sepsis identified in the emergency room was termed community-onset, while sepsis diagnosed in hospitalized patients was defined as hospital-onset sepsis.

“Time zero” was defined as either the time of triage in the emergency room or the time identified by the rapid response team in the ward.

### Identification of AKI

Serum creatinine levels at time zero, on ICU days 1–3 and 7, and on the last day of ICU admission were recorded in the database. Since creatinine levels before study enrollment were not available, the baseline creatinine level was estimated as follows: 1) In patients without chronic kidney disease (CKD), the estimated glomerular filtration rate (eGFR) was assumed to be 75 mL/min/1.73 m^2^, and the baseline creatinine value was calculated using the Modification of Diet in Renal Disease equation [14]. 2) For patients with CKD, the baseline creatinine value was determined using the lowest serum creatinine level recorded at six time points in the database: time zero, ICU days 1–3 and 7, and the last day of ICU admission.

The creatinine criteria of the KDIGO definition were employed to identify AKI [11]. We did not use urine output criteria to identify AKI since the database only provided daily urine output measurements during the ICU stay rather than hourly urine output data.

### Identification of SA-AKI

The development of AKI by ICU day 7 was defined as SA-AKI. Given that the median time from diagnosis of sepsis to ICU admission was 7.2 h (interquartile range [IQR], 4.5–13.5 h), it was deemed appropriate to categorize AKI diagnosed by ICU day 7 as SA-AKI. The severity of SA-AKI was determined by the highest AKI stage diagnosed by ICU day 7.

### Statistical analysis

Baseline characteristics are presented as means (standard deviations [SDs]) or medians (IQRs) for continuous variables and as numbers (percentages) for categorical variables. The χ^2^ test (for categorical variables), Wilcoxon rank sum test (for nonparametric continuous variables), and t-test (for parametric continuous variables) were performed to investigate between-group differences. Differences in time-to-event distributions were evaluated using the log-rank test and visualized using Kaplan–Meier curves. To examine characteristics associated with severe SA-AKI and in-hospital mortality, we used a multilevel logistic regression model, accounting for the variability across different hospitals [15]. The variables in the multivariable logistic model were selected by least absolute shrinkage and selection operator (LASSO) regression (e-Method 3) [16].

Given the study’s prospective observational design and the minimal presence of missing data, no imputations were performed for missing data. Analyses were performed using R statistical software, version 4.2.3 (R Project for Statistical Computing). Statistical significance for the two-sided *P *value was set at < 0.05.

## Results

### Patient characteristics

Of the 13,827 patients diagnosed with sepsis during the study period, 5293 were admitted to the ICU. After excluding 191 patients (184 patients requiring chronic dialysis for end-stage renal disease and 7 patients with time zero of sepsis documented after ICU admission), the final cohort comprised 5100 patients (mean age [SD], 71.2 years [13.8]; 2960 males [58.0%]) (Fig. 1). Patients diagnosed with sepsis but not admitted to the ICU are described in Supplementary e-Fig. 1 and e-Table 2.

In the comparison of characteristics between patients with and without SA-AKI among the 5,100 patients admitted to ICUs, community-onset sepsis was more frequent in patients with SA-AKI than in those without SA-AKI. Respiratory infections were less prevalent, whereas abdominal, urinary tract, and unknown sources of infection were more common in patients with SA-AKI than in those without SA-AKI. The incidence of bacteremia was higher in patients with SA-AKI with a higher incidence of Gram-negative bacteremia (Table 1 and Supplementary e-Table 3).

**Table 1:** Baseline characteristics of the study population

Variables	All patients (n = 5100)	With SA-AKI (n = 3177)	Without SA-AKI (n = 1923)	*P* value
Age, year	71.2 ± 13.8	72.2 ± 13.2	69.6 ± 14.4	< 0.001
Sex				< 0.001
Male	2960 (58.0)	1777 (55.9)	1183 (61.5)	
Female	2140 (42.0)	1400 (44.1)	740 (38.5)	
Body mass index (kg/m^2^)	22.6 ± 30.5	23.4 ± 38.5	21.2 ± 4.4	< 0.001
Comorbidities				
Diabetes mellitus	1834 (36.0)	1261 (39.7)	573 (29.8)	< 0.001
Cardiovascular disease	957 (18.8)	661 (20.8)	296 (15.4)	< 0.001
Chronic lung disease	535 (10.5)	300 (9.4)	235 (12.2)	0.002
Chronic kidney disease	522 (10.2)	404 (12.7)	118 (6.1)	< 0.001
Solid malignant tumors	1480 (29.0)	871 (27.4)	609 (31.7)	0.001
Hematological malignancies	375 (7.4)	226 (7.1)	149 (7.7)	0.432
Immunocompromised	204 (4.0)	133 (4.2)	71 (3.7)	0.424
Charlson Comorbidity Index	5.3 ± 2.5	5.4 ± 2.5	5.1 ± 2.5	< 0.001
Clinical frailty score	5.2 ± 2.2	5.1 ± 2.1	5.3 ± 2.2	< 0.001
1 ~ 3	1288 (25.3)	798 (25.1)	490 (25.5)	< 0.001
4 ~ 6	1962 (38.5)	1321 (41.6)	641 (33.3)	
7 ~ 9	1850 (36.3)	1058 (33.3)	792 (41.2)	
Characteristics of infection				
Type of infection				< 0.001
Community-onset sepsis	3921 (76.9)	2493 (78.5)	1428 (74.3)	
Hospital-onset sepsis	1179 (23.1)	684 (21.5)	495 (25.7)	
Surgical admission	395 (7.7)	209 (6.6)	186 (9.7)	< 0.001
Site of infection^*^				
Respiratory	2293 (45.0)	1252 (39.4)	1041 (54.1)	< 0.001
Abdominal	1433 (28.1)	949 (29.9)	484 (25.2)	< 0.001
Urinary tract	1070 (21.0)	800 (25.2)	270 (14.0)	< 0.001
Skin and soft tissue	203 (4.0)	130 (4.1)	73 (3.8)	0.653
Others	99 (1.9)	52 (1.6)	47 (2.4)	0.055
Unknown	443 (8.7)	305 (9.6)	138 (7.2)	0.003
Bacteremia	2379 (46.6)	1586 (49.9)	793 (41.2)	< 0.001
Gram positive bacteremia	709 (13.9)	454 (14.3)	255 (13.3)	0.323
Gram negative bacteremia	1995 (39.1)	1343 (42.3)	652 (33.9)	< 0.001
Multidrug-resistant bacteria^†^	677 (13.3)	440 (13.8)	237 (12.3)	0.13
Characteristics at diagnosis of sepsis				
Vital signs				
Mean blood pressure (mmHg)	70.0 ± 21.0	67.2 ± 20.0	74.7 ± 21.8	< 0.001
Heart rate (/min)	107.7 ± 26.7	106.1 ± 27.1	110.4 ± 25.7	< 0.001
Temperature (°C)	37.2 ± 1.3	37.1 ± 1.4	37.4 ± 1.2	< 0.001
Laboratory values				
Lactate (mmol/L)	4.6 ± 3.8	5.2 ± 4.1	3.6 ± 3.0	< 0.001
eGFR at time zero (mL/min/1.73 m^2^)	54.6 ± 51.2	32.2 ± 22.9	91.8 ± 62.4	< 0.001
SOFA score	7.3 ± 3.1	8.1 ± 3.1	6.0 ± 2.6	< 0.001
Renal SOFA score	1.2 ± 1.2	1.7 ± 1.1	0.2 ± 0.5	< 0.001
Non-renal SOFA score	6.2 ± 2.8	6.4 ± 2.9	5.8 ± 2.6	< 0.001
Characteristics during ICU stay				
SOFA score on ICU day 1	9.7 ± 3.8	10.8 ± 3.7	8.0 ± 3.3	< 0.001
eGFR at ICU admission (mL/min/1.73 m^2^)	57.6 ± 61.6	32.7 ± 22.2	99.0 ± 81.0	< 0.001
SAPS 3 on ICU day 1	73.9 ± 15.7	76.9 ± 15.5	68.9 ± 14.7	< 0.001
Septic shock on ICU day 1	2456 (48.7)	1650 (52.4)	806 (42.6)	< 0.001
Invasive ventilation on ICU day 1	2323 (45.5)	1514 (47.7)	809 (42.1)	< 0.001
Time from diagnosis of sepsis to ICU admission (hr)	7.2 (4.5—13.5)	7.2 (4.5—13.3)	7.3 (4.4—13.8)	0.851

Data are presented as n (%), mean ± standard deviation, or median (interquartile range)

eGFR, estimated glomerular filtration rate; ICU, intensive care unit; SA-AKI, sepsis-associated acute kidney injury; SAPS, Simplified Acute Physiology Score; SOFA, Sequential Organ Failure Assessment

*Mutually nonexclusive

†Detailed explanation in e-Method 2

### SA-AKI incidence and trajectory

Overall, SA-AKI developed in 3177 (62.3%) patients. Of these, 613 patients (19.3%) had stage 1 SA-AKI, 721 (22.7%) had stage 2, and 1843 (58.0%) had stage 3.

Among the patients diagnosed with SA-AKI, 2506 (78.9%) developed SA-AKI at diagnosis of sepsis, and 2828 (89.0%) developed SA-AKI by ICU day 1. Additionally, among all patients with SA-AKI, 2008 (63.2%) reached their highest stage of SA-AKI simultaneously with the diagnosis of sepsis, and the number increased to 2571 (80.9%) by ICU day 1 (Supplementary e-Table 4). The mean serum creatinine level of patients with SA-AKI peaked on ICU day 1 and decreased thereafter in all stages of SA-AKI (Supplementary e-Fig. 2). The trajectory of renal function status is shown in Supplementary e-Fig. 3 and e-Table 5, demonstrating that the renal recovery rate on the last day of ICU was 84.8% for SA-AKI stage 1, with the rate decreasing as the stage increased. For SA-AKI stage 3, more than half of the patients remained in stage 3 on the last day of ICU.

### Outcomes according to SA-AKI stages

**Fig. 2:**
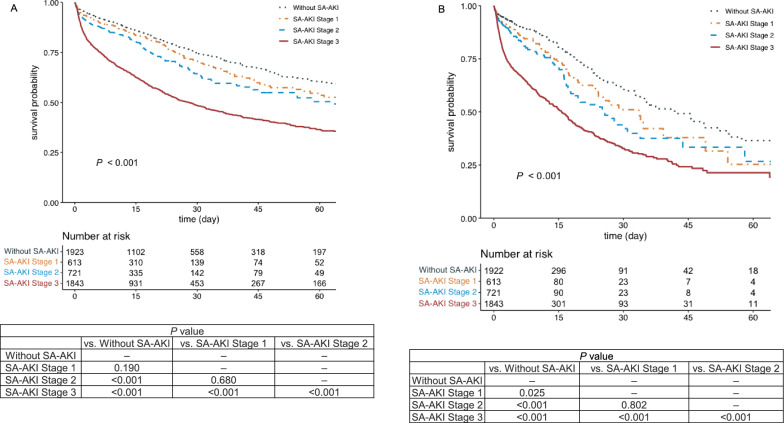
Kaplan–Meier survival curves according to the sepsis-associated acute kidney injury stage. **A** Kaplan–Meier survival curves for in-hospital mortality. **B** Kaplan–Meier survival curves for ICU mortality. ICU, intensive care unit; SA-AKI, sepsis-associated acute kidney injury

The risk of in-hospital mortality for patients with stage 3 SA-AKI was significantly higher than for those with lower stages (*P* < 0.001 for all comparisons). While stage 2 SA-AKI also increased the risk of in-hospital mortality compared to patients without SA-AKI (*P* < 0.001), there was no statistical difference when compared to stage 1. Additionally, there was no statistically significant difference in in-hospital mortality risk between patients with stage 1 SA-AKI and those without SA-AKI. Regarding ICU mortality, the risk of ICU mortality increased with higher SA-AKI stages, except between those with stage 1 SA-AKI and those with stage 2 SA-AKI without statistical significance (Fig. 2; Supplementary e-Table 6).

After controlling for confounders, only stage 3 SA-AKI showed a significant increase in the risk of in-hospital mortality compared with those without SA-AKI. Regarding ICU mortality, both stages 2 and 3 of SA-AKI were associated with a higher risk of ICU mortality compared to those without SA-AKI (Supplementary e-Fig. 4).

### Factors associated with severe SA-AKI

We examined factors associated with severe SA-AKI (Table 2). Demographic factors independently associated with severe SA-AKI included older age, female sex, and the presence of comorbidities such as diabetes mellitus, cardiovascular disease, and CKD. Of the variables related to infection, urinary tract infection was associated with an increased risk of severe SA-AKI. Conversely, respiratory infection and administration of antibiotics before time zero were associated with a decreased risk of severe SA-AKI.

**Table 2:** Results of bivariate and multivariate logistic regression analyses for severe SA-AKI

Variables	Bivariate analysis			Multivariable logistic regression analysis^*,†^	
	Severe SA-AKI (n = 2564)	Without SA-AKI or SA-AKI stage 1 (n = 2530)	*P* value	Adjusted odds ratio (95% CI)	*P* value
Age	72.0 ± 13.4	70.5 ± 14.1	< 0.001	1.01 (1.00–1.01)	0.026
Sex, male	1414 (55.1)	1546 (61.0)	< 0.001	0.87 (0.77–0.98)	0.023
Body mass index	22.9 ± 4.6	22.3 ± 43.0	< 0.001		
Clinical frailty score			< 0.001		
1 ~ 3	641 (25.0)	647 (25.5)			
4 ~ 6	1081 (42.2)	881 (34.7)			
7 ~ 9	842 (32.8)	1008 (39.7)			
Comorbidity					
Diabetes mellitus	1037 (40.4)	797 (31.4)	< 0.001	1.30 (1.15–1.47)	< 0.001
Cardiovascular disease	549 (21.4)	408 (16.1)	< 0.001	1.24 (1.07–1.45)	0.005
Chronic lung disease	242 (9.4)	293 (11.6)	0.016		
Chronic kidney disease	364 (14.2)	158 (6.2)	< 0.001	2.37 (1.93–2.92)	< 0.001
Solid malignant tumors	713 (27.8)	767 (30.2)	0.059		
Hematological malignancies	185 (7.2)	190 (7.5)	0.745		
Immunocompromised	115 (4.5)	89 (3.5)	0.088		
Type of infection			0.032		
Hospital-onset sepsis	560 (21.8)	619 (24.4)			
Community-onset sepsis	2004 (78.2)	1917 (75.6)			
Site of infection^‡^					
Respiratory	1000 (39.0)	1293 (51.0)	< 0.001	0.66 (0.58–0.75)	< 0.001
Abdominal	762 (29.7)	671 (26.5)	0.011		
Urinary tract	670 (26.1)	400 (15.8)	< 0.001	1.52 (1.30–1.78)	< 0.001
Bacteremia					
Gram positive bacteremia	391 (15.2)	318 (12.5)	0.006		
Gram negative bacteremia	1101 (42.9)	894 (35.3)	< 0.001		
Multidrug-resistant bacteria^§^	350 (13.7)	327 (12.9)	0.451		
Antibiotics before time zero	756 (29.5)	855 (33.7)	0.001	0.76 (0.67–0.88)	< 0.001

Data are presented as n (%) or mean ± standard deviation

CI, confidence interval; SA-AKI, sepsis-associated acute kidney injury; SOFA, Sequential Organ Failure Assessment

^*^Variables included in the multivariate model were selected through the least absolute shrinkage and selection operator (LASSO) regression for variables in the bivariate analysis

^†^Multilevel logistic regression model was adopted to adjust variability between hospitals

^‡^Mutually nonexclusive

^§^Detailed explanation e-Method 2

### Factors associated with in-hospital mortality

Based on the Kaplan–Meier survival analysis for in-hospital mortality, which revealed no difference between patients without SA-AKI and those with stage 1 SA-AKI but significant differences compared to those with stages 2 and 3, we classified stages 2 and 3 as severe SA-AKI. We then investigated the factors associated with in-hospital mortality in patients with severe SA-AKI (Table 3).

**Table 3:** Results of bivariate and multivariate logistic regression analyses for in-hospital mortality in patients with severe SA-AKI

Variables		Bivariate analysis			Multivariable Logistic Regression Analysis^*,†^	
		Survival (n = 1,365)	Non-survival (n = 1,199)	*P* value	Adjusted odds ratio (95% CI)	*P* value
Age		71.7 ± 13.7	72.3 ± 13.0	0.535	1.01 (1.00–1.02)	0.014
Sex, male		704 (51.6)	710 (59.2)	< 0.001	0.92 (0.76–1.12)	0.419
Body mass index		23.0 ± 4.6	22.8 ± 4.5	0.46		
Clinical frailty score				< 0.001		
1–3		384 (28.1)	257 (21.4)			
4–6		568 (41.6)	513 (42.8)			
7–9		413 (30.3)	429 (35.8)			
Comorbidity						
Diabetes mellitus		589 (43.2)	448 (37.4)	0.003		
Cardiovascular disease		280 (20.5)	269 (22.4)	0.256		
Chronic lung disease		109 (8.0)	133 (11.1)	0.009		
Chronic kidney disease		174 (12.7)	190 (15.8)	0.029	1.35 (1.04–1.77)	0.025
Solid malignant tumors		329 (24.1)	384 (32.0)	< 0.001		
Hematological malignancies		60 (4.4)	125 (10.4)	< 0.001	1.38 (0.94–2.02)	0.097
Immunocompromised		52 (3.8)	63 (5.3)	0.095		
SOFA score on ICU day1		9.9 ± 3.3	12.7 ± 3.5	< 0.001	1.16 (1.12–1.20)	< 0.001
SAPS3 on ICU day 1		72.8 ± 13.1	84.7 ± 15.6	< 0.001	1.04 (1.03–1.05)	< 0.001
Septic shock on ICU day 1		662 (49.0)	659 (55.1)	0.002		
Type of Infection				< 0.001		
Hospital-onset sepsis		236 (17.3)	324 (27.0)			
Community-onset sepsis		1129 (82.7)	875 (73.0)			
Site of infection^‡^						
Respiratory		467 (34.2)	533 (44.5)	< 0.001		
Abdominal		386 (28.3)	376 (31.4)	0.097		
Urinary tract		474 (34.7)	196 (16.3)	< 0.001	0.39 (0.31–0.49)	< 0.001
Bacterial blood sepsis						
Gram positive blood sepsis		189 (13.8)	202 (16.8)	0.04		
Gram negative blood sepsis		623 (45.6)	478 (39.9)	0.004		
Multidrug-resistant bacteria^§^		183 (13.4)	167 (13.9)	0.744		
Antibiotics before time zero		343 (25.1)	413 (34.4)	< 0.001		
Appropriateness of empirical antibiotics^§^		1230 (90.8)	1029 (86.7)	0.001	0.63 (0.47–0.85)	0.003
1 Hr bundle compliance, %						
Broad spectrum antibiotics		337 (24.7)	332 (27.7)	0.09		
Fluid resuscitation		1,177 (86.2)	928 (77.4)	< 0.001	0.62 (0.48–0.79)	< 0.001
Vasopressors		816 (59.8)	675 (56.3)	0.081		
Use of nephrotoxic antibiotics^§^		243 (17.8)	302 (25.2)	< 0.001		
Adjunctive steroid		275 (20.1)	360 (30.0)	< 0.001		
Source control (either surgical or non-surgical)		256 (18.8)	143 (11.9)	< 0.001	0.53 (0.41–0.69)	< 0.001
Vasopressors on ICU day 1		1090 (79.9)	1072 (89.4)	< 0.001		
Invasive mechanical ventilation on ICU day 1		498 (36.5)	773 (64.5)	< 0.001		

Data are presented as n (%) or mean ± standard deviation

CI, confidence interval; ICU, intensive care unit

*Variables included in the multivariate model were selected through the least absolute shrinkage and selection operator (LASSO) regression for variables in the bivariate analysis

†Multilevel logistic regression model was adopted to adjust variability between hospitals

‡Mutually nonexclusive

§Detailed explanation in e-Method 2

In patients with severe SA-AKI, age, presence of CKD, and elevated SOFA and Simplified Acute Physiology Scores 3 were associated with increased risk of in-hospital mortality. Urinary tract infection, appropriateness of empirical antibiotics, adherence to the fluid resuscitation component of the one-hour sepsis bundle, and infection source control were associated with a decreased risk of in-hospital mortality. The results of the logistic regression analysis for in-hospital mortality in patients without SA-AKI and with stage 1 SA-AKI are shown in Supplementary e-Table 7.

### Sensitivity analysis

We performed three sensitivity analyses for the incidence of SA-AKI and the association between the fluid resuscitation component of the one-hour sepsis bundle and in-hospital mortality using different methods for assuming the baseline creatinine level: 1) back-calculating the serum creatinine value assuming the eGFR is 65 mL/min/1.73 m^2^ in patients without CKD; 2) back-calculating the serum creatinine value assuming the eGFR is 70 mL/min/1.73 m^2^ in patients without CKD; and 3) back-calculating the serum creatinine assuming the eGFR is 75 mL/min/1.73 m^2^ for all patients in the cohort. The incidence of SA-AKI was similar in all three sensitivity analyses. Adherence to the fluid resuscitation component was consistently associated with a decreased risk of in-hospital mortality in the severe SA-AKI group but not in those without SA-AKI or with stage 1 SA-AKI (Supplementary e-Table 8).

## Discussion

Of the 5100 patients who were admitted to the ICU with a diagnosis of sepsis, SA-AKI was confirmed in 62.3%: 19.3% with stage 1, 22.7% with stage 2, and 58.0% with stage 3. The majority of SA-AKI cases occurred early in the course of a sepsis diagnosis, with 89.0% developing within ICU day 1. Severe SA-AKI was associated with an increased risk of crude short-term mortality. The appropriateness of empirical antibiotics, adherence to the fluid resuscitation component of the one-hour sepsis bundle, and infection source control were associated with a decreased risk of mortality in severe SA-AKI.

Our study can be compared with White et al.’s study [17], which recently reported the epidemiology of SA-AKI based on the SA-AKI definition of the ADQI 28 workgroup. The study by White et al. involved 84,528 ICU admissions, with 27.9% (23,555) of patients diagnosed with sepsis, and 57.1% (13,451) of these patients meeting the SA-AKI criteria. Among the patients with SA-AKI, 7239 (54.0%) had stage 1 SA-AKI, 3387 (25%) had stage 2, and 2825 (21%) had stage 3 SA-AKI. Stage 2 has 3387 patients. The in-hospital mortality for patients with SA-AKI by stage were 14.4% (1014/7239) for stage 1, 20.7% (701/3387) for stage 2, and 22.6% (639/2825) for stage 3. Both our study and White’s study highlighted that SA-AKI is a common and increasingly prevalent problem in the ICU, typically diagnosed within a day of ICU admission.

The methodological differences between our study and that of White et al. can be explained as follows: First, the approaches to estimating baseline creatinine levels differed. A shared limitation of both studies is the lack of baseline creatinine level data in all patients. Consequently, both studies estimated creatinine levels by assuming an estimated glomerular filtration rate (eGFR) of 75 mL/min/1.73m2. In White’s study, this rule was uniformly applied to all patients, whereas in our study, the nadir creatinine value during the first 7 days of the ICU stay was used as the baseline creatinine for patients with CKD. Second, the criteria for defining AKI were different. In White’s study, routinely collected electronic medical records were retrospectively utilized to define AKI according to the serum creatinine level and urine output criteria of the KDIGO definition. Approximately 20% of urine output records were either zero or missing, and in such cases, the average value over preceding intervals was used. As a result, patients without urine output measurements might have been inaccurately classified as having low urine output. In contrast, our study applied only the creatinine-based criteria from the KDIGO guidelines and did not incorporate urine output criteria into the AKI definition.

Using only serum creatinine levels to diagnose SA-AKI might underestimate SA-AKI cases by failing to identify patients whose AKI was detected solely through urine output criteria. However, we believe the results of our study remain significantly relevant for the following reasons: First, exploring the epidemiology of SA-AKI based on creatinine-level criteria mirrors the real-world sepsis scenario. Hourly urine output can only be monitored in patients with urinary catheters. Urine output data frequently goes unrecorded in busy clinical settings, particularly in emergency departments. For these reasons, many studies using observational datasets or registry databases have omitted the urine output’s contribution in the definition of AKIs [18, 19]. Second, oliguria can arise as a physiological response to various causes without a decrease in glomerular filtration rates, especially in critically ill patients. Such factors include neurohormonal activation from pain, stress, and hypotension; reduced venous return from positive pressure ventilation; and sodium and fluid retention from steroid use [20, 21]. While oliguria is associated with poor outcomes [22, 23], many authors do not consider it strongly specific to AKI [21, 24]. Third, AKI cases that only meet the urine output criteria tend to have better outcomes than those meeting the creatinine-level criteria, underscoring the differential impact of these diagnostic criteria on patient outcomes [23]. Therefore, it is expected that the creatinine level criteria will appropriately categorize most SA-AKI cases, which significantly impact patients’ clinical outcomes.

In our study, the proportion of stage 3 SA-AKI was higher compared to previous research, which reported 20–30% for SA-AKI [17, 25]. We attribute this to the following two aspects: First, the disease severity of the patient population in our study was higher. Second, there are differences in the timeframes used to define the severity of SA-AKI in studies. In the Protocolized Care for Early Septic Shock trial, the severity of AKI was defined based on the maximum stage until ICU discharge or 72 h from study enrollment [25]. White’s study defined the severity of AKI based on the severity at the time of SA-AKI diagnosis [17]. Whereas we defined the severity of AKI as the maximum stage within 7 days from time zero. When assessing severity at the time of SA-AKI diagnosis in our study, the stage distribution was as follows: 882 patients (27.8%) had stage 1933 (29.4%) had stage 2, and 1362 patients (42.9%) had stage 3.

Stage 1 SA-AKI did not lead to an increase in crude in-hospital mortality compared to patients without SA-AKI. This might be due to the fact that most cases of stage 1 SA-AKI tend to resolve as sepsis improves; on the last day of ICU, 84.8% of patients with stage 1 SA-AKI had recovered from their SA-AKI (Supplementary e-Table 5). Previous literature also supports this, indicating that transient AKI does not worsen mortality rates [26, 27]. However, it should be noted that our study did not examine long-term outcomes. Therefore, the interpretation that stage 1 SA-AKI does not affect mortality is limited to short-term outcomes, and caution is required when interpreting these results.

In the multivariable logistic regression analysis of in-hospital mortality in patients with severe SA-AKI, urinary tract infection was associated with decreased mortality, possibly owing to the ease of controlling the source of the infection [28–30]. Regarding the one-hour sepsis bundle compliance, adherence to the fluid resuscitation component was independently associated with a decreased risk of in-hospital mortality. This result emphasizes the importance of initial fluid resuscitation to restore tissue perfusion, especially in patients with severe SA-AKI. This could be supported by the fact that a reduction in global renal blood flow plays a predominant role in the pathophysiology of SA-AKI, although it is not the sole mechanism involved [31].

For sepsis-induced hypotension, fluid administration is commonly categorized into three distinct phases: the first hour from time zero (initial resuscitation), followed by the subsequent 24 h (early resuscitation), and beyond that period. The significance of initial resuscitation, aligning with the one-hour sepsis bundle for fluid resuscitation, was previously discussed. The volume of fluid administered during early resuscitation was not associated with mortality, aligning with the findings of recent large-scale RCTs (Supplementary e-Fig. 5) [32, 33]. However, fluid overload beyond the early resuscitation phase was found to be associated with higher mortality (Supplementary e-Fig. 6).

### Strengths and limitations

Our study had the following strengths: First, this was a multicenter observational cohort that prospectively defined sepsis, ensuring the accuracy of both the diagnosis and its timing, thereby reflecting real-world sepsis scenarios. Secondly, by verifying CKD history and estimating baseline creatinine differently based on CKD presence, we could reduce the risk of over-diagnosing AKI, often seen when estimating baseline creatinine based on an eGFR of 75 mL/min/1.73 m^2^ in patients with CKD.

Nevertheless, our study had several limitations. First, we did not use urine output criteria to identify AKI, which may have led to an underestimation of AKI incidence and a delay in diagnosis. However, defining AKI based solely on creatinine-level criteria reflects a ‘real-life snapshot of AKI and sepsis,’ especially given the challenges of obtaining accurate hourly urine output data. This is particularly relevant in critically ill patients, where urine output criteria may misleadingly attribute various physiological renal adaptations to AKI. Second, baseline creatinine levels were not available for all patients. Nevertheless, by conducting sensitivity analyses that involved adjustments to the assumed eGFRs, we confirmed that the incidence rates remained similar. Third, since time zero is defined as the time of emergency room triage in community onset sepsis, the actual onset of sepsis may have occurred earlier.

## Conclusions

SA-AKI developed in 62.3% of patients admitted to the ICU with a diagnosis of sepsis. Most SA-AKI cases occurred at sepsis diagnosis and reached their highest SA-AKI stages by the first day of ICU admission. Severe SA-AKI was associated with an increased risk of short-term mortality. Based on our findings, following the initial fluid resuscitation component of a one-hour sepsis bundle can potentially improve outcomes in patients with severe SA-AKI. This study provides high-quality epidemiological evidence on SA-AKI in patients admitted to ICUs. However, further research is needed that applies both urine output and creatinine-based criteria to identify SA-AKI and assess the relationship between each criterion and patient outcomes.

## Supplementary Information

Additional file1.

## Abbreviations

ADQI: Acute Disease Quality Initiative
AKI: Acute kidney injury
CKD: Chronic kidney disease
eGFR: Estimated glomerular filtration rate
ICU: Intensive care unit
IQR: Interquartile range
KDIGO: Kidney Disease Improving Global Outcomes
KSA: Korean Sepsis Alliance
LASSO: Least absolute shrinkage and selection operator
SA-AKI: Sepsis-associated acute kidney injury
SD: Standard deviation
STROBE: Strengthening the Reporting of Observational Studies in Epidemiology
SOFA: Sequential Organ Failure Assessment

## Declarations

### Availability of data and materials

No datasets were generated or analysed during the current study. The datasets used and/or analyzed during the current study are available from the corresponding author upon reasonable request.
