# Supplementary material for: Epidemiology of sepsis-associated acute kidney injury in critically ill patients: a multicenter, prospective, observational cohort study in South Korea
Source: Crit Care. 2024 Nov 24;28:383. doi: 10.1186/s13054-024-05167-9 (PMC11587587; doi:10.1186/s13054-024-05167-9)
Supplement: Supplementary file 1 — Additional file1. [file 13054_2024_5167_MOESM1_ESM.docx]

Epidemiology of sepsis-associated acute kidney injury in critically ill patients: a multicenter, prospective, observational cohort study in South Korea

e-Method 1. Detailed information on Korean Sepsis Alliance (KSA) registry

e-Method 2. Detailed information on data collection

e-Method 3. Detailed explanation of variable selection for multivariable logistic models:

e-Figure 1. Patient trajectory for those diagnosed with sepsis but not admitted to the ICU

e-Figure 2. Changes in the mean serum creatinine level (mg/dL) according to the SA-AKI stage (patients classified as stage 3 based on CRRT were excluded)

e-Figure 3. Trajectory of AKI severity in patients with SA-AKI

e-Figure 3. Changes in the mean serum creatinine level according to the severity of SA-AKI

e-Figure 4. Risk difference for in-hospital mortality and ICU mortality according to the severity of SA-AKI relative to those without SA-AKI.

e-Figure 5. Estimated odds ratio for in-hospital mortality and ICU mortality based on the cumulative dose of fluid intake from time zero to ICU day 1

e-Figure 6. Estimated odds ratio for in-hospital mortality and ICU mortality based on the cumulative dose of fluid intake from time zero to ICU day 3

e-Table 1. Missing values

e-Table 2. Baseline characteristics of patients admitted to the general ward, categorized by their AKI status at time zero

e-Table 3. Baseline characteristics of the study population by SA-AKI stage

e-Table 4. Timing of SA-AKI diagnosis

e-Table 5. The persistence of AKI and renal function on the last day of ICU

e-Table 6. Outcomes categorized by the severity of sepsis-associated acute kidney injury

e-Tabel 7. Results of bivariate and multivariable logistic regression analyses for in-hospital mortality in patients without SA-AKI and SA-AKI stage 1

e-Table 8. Sensitivity analysis: SA-AKI incidence and impact of 1-hour fluid resuscitation using three different methods for assuming the baseline creatinine value

e-Method 1. Detailed information on Korean Sepsis Alliance (KSA) registry

In 2019, the Korean Sepsis Alliance (KSA) was founded as part of a policy research initiative focused on improving the management of sepsis patients, spearheaded by the Korea Centers for Disease Control and Prevention (KCDC). The KSA, in collaboration on with the Korean Society of Critical Care Medicine (KSCCM), has created the KSA registry–a web-based platform for sepsis data involving participation from tertiary or university-affiliated hospitals in South Korea.

Since September 2019, the KSA registry has been prospectively collecting data on sepsis patients from participating hospitals. After data accumulates over a certain period, Asan Medical Center evaluates the data's quality for completeness and logical errors before releasing the data. At the time of this study, the KSA data has been released in four phases: first dataset from September 2019 to February 2020, the second dataset from September 2019 to December 2020, the third dataset from September 2019 to December 2021, and the fourth dataset from September 2019 to December 2022. This study was performed using the fourth data released.

e-Method 2. Detailed information on data collection

The following data were collected: (1) demographic characteristic, including age, sex, body mass index, comorbidities, Charlson Comorbidity Index, clinical frailty score; (2) microbiological characteristics, including the type of infection, primary site, and pathogen; (3) laboratory data at the time of sepsis diagnosis (values closest to time zero), on intensive care unit (ICU) days 1–3 and 7, and on the last ICU day; (4) vital signs at time zero, on ICU days 1–3 and 7, and on the last ICU day; (5) treatment-related data, including compliance with the one-hour sepsis bundle elements, adjunctive corticosteroid treatment, source control of infection, antibiotic therapy, daily fluid balance, vasopressor and inotrope therapy, invasive mechanical ventilation, and renal replacement therapy during the ICU stay; and (6) clinical outcomes, including ICU and in-hospital mortalities, hospital length of stay and ICU length of stay.

The definitions of comorcidities were based on those from the MOSAIC II study [1]. The definitions of comorcidities and several variables are elaborated below:

**Diabetes mellitus**: Diagnosed with DM, regardless of the type.

**Cardiovascular disease**: Conditions including ischemic heart disease (IHD) and heart failure. Hypertension is not included.

**Chronic lung disease**: Conditions including chronic obstructive pulmonary disease (COPD), asthma, bronchiectasis, post-tuberculosis related lung disease and interstitial lung disease (ILD). Exclude primary or secondary lung malignancy. Patients who are undergoing treatment for tuberculosis or non-tuberculosis mycobacterium (NTM) prior to ICU admission should be included in this category.

**Chronic kidney disease**: Kidney damage > 3 months (abnormal blood/urine composition or radiological renal abnormalities or glomerular filtration rate < 60mL/min/1.73m2), excluding renal cell carcinoma.

**Solid malignant tumors**: This would include malignancies such as breast, colon, lung, prostate, skin, etc

**Hematological malignancies**: This would include, but not limited to leukemia, lymphoma and multiple myeloma.

**Immunosuppression**: Patients on long term steroids or other immunosuppressants (excluding patients with hematological conditions or other malignancies)

**Multidrug-resistant bacteria**: either Methicillin Resistant Staphylococcus aureus, Vancomycin Resistant Enterococcus, ESBL producing Enterobacteriaceae, Carbapenem Resistant Enterobacteriaceae, Carbapenem Resistant Pseudomonas aeruginosa, Carbapenem Resistant Acinetobacter baumannii.

**Appropriateness of empirical antibiotics**: the suitability of broad-spectrum antibiotics administered as part of the one-hour bundle care. When antibiotics susceptibility results of culture are available, appropriateness of prescribed antibiotics is evaluated based on the result. In the absence of antibiotics susceptibility results, appropriateness is determined by adherence to the general practice guidelines of empirical antibiotics.

**Use of nephrotoxic antibiotics:** Patients who have been treated with any of the following antibiotics; colistin, glycopeptides, or aminoglycosides.

e-Method 3. Detailed explanation of variable selection for multivariable logistic models:

The variables in the multivariable logistic models were selected by least absolute shrinkage and selection operator (LASSO) regression. LASSO regression is a variable selection method that aims to identify the smallest subset of the most important variables, which minimizes the model’s prediction error. The optimal value of the tuning constant (λ) was determined using ten-fold cross-validations with 1 standard error. Since the majority of SA-AKIs were identified at time zero, the variables included in the LASSO regression for selecting predictors of severe SA-AKI were those established before time zero (listed in the bivariate analysis in Table 2). In the analysis of in-hospital mortality, treatment-related variables were also considered, in addition to those utilized for analyzing severe SA-AKI development (listed in the bivariate analysis in Table 3)

e-Figure 1. Patient trajectory for those diagnosed with sepsis but not admitted to the ICU


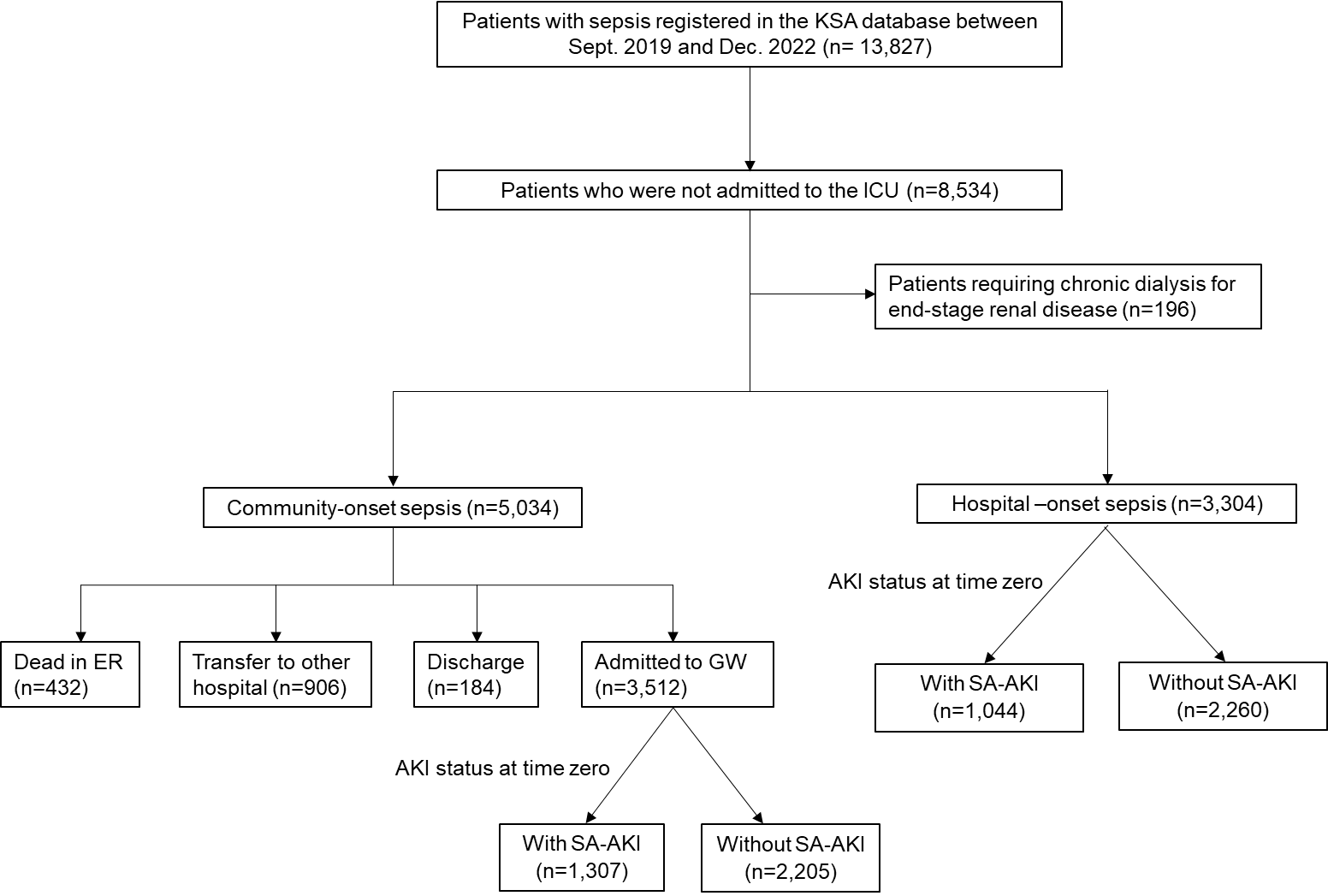


AKI, acute kidney injury; ER, emergency room; GW, general ward; ICU, intensive care unit; KSA, Korean Sepsis Alliance; SA-AKI, sepsis-associated acute kidney injury

e-Figure 2. Changes in the mean serum creatinine level (mg/dL) according to the SA-AKI stage (patients classified as stage 3 based on CRRT were excluded)

(B) Changes in the mean serum creatinine in patients with CKD

(A) Changes in the mean serum creatinine in patients without CKD


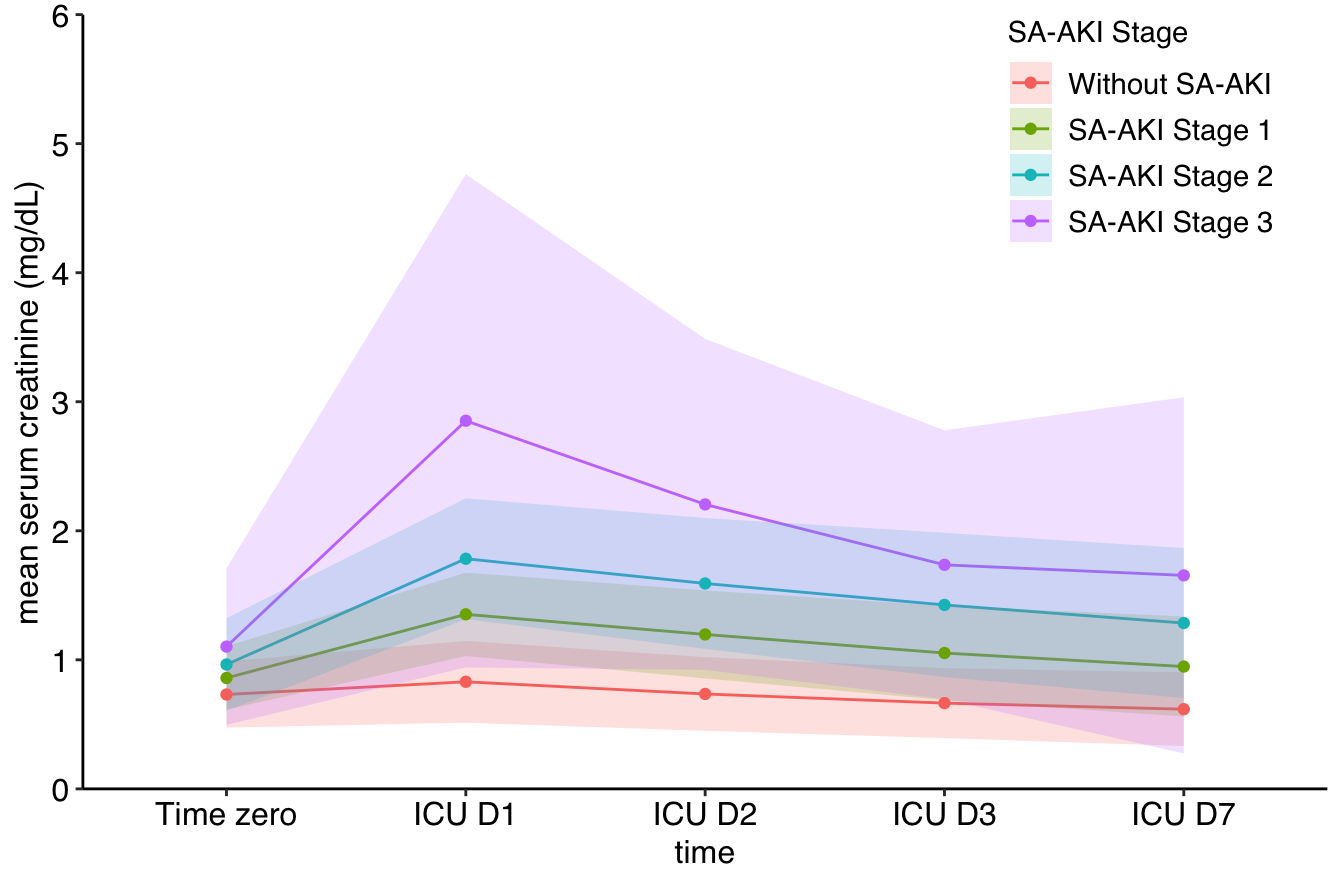

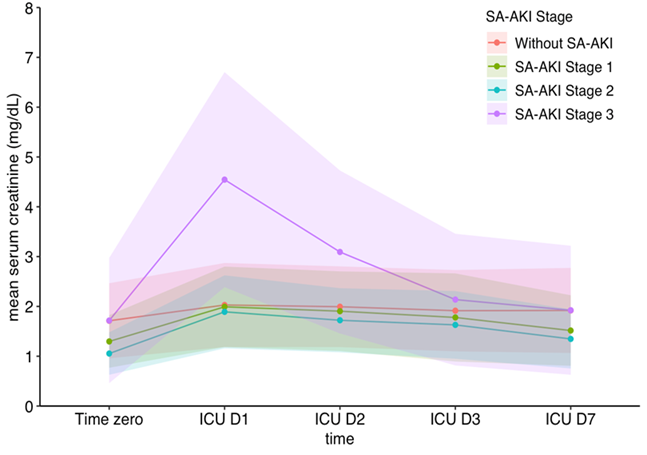


| Severity of  SA-AKI | Non-CKD | | | | | CKD | | | | |
| --- | --- | --- | --- | --- | --- | --- | --- | --- | --- | --- |
|  | Time zero | Day 1 | Day 2 | Day 3 | Day 7 | Time zero | Day 1 | Day 2 | Day 3 | Day 7 |
| Without  SA-AKI | 0.6 ± 0.2  (n=1805) | 0.8 ± 0.3  (n=1753) | 0.7 ± 0.3  (n=1773) | 0.7 ± 0.3  (n=1768) | 0.6 ± 0.3  (n=1272) | 1.6 ± 0.7  (n=118) | 2.0 ± 0.8  (n=118) | 2.0 ± 0.8  (n=117) | 1.9 ± 0.8  (n=117) | 1.9 ± 0.9  (n=81) |
| SA-AKI  Stage 1 | 0.8 ± 0.3  (n=573) | 1.4 ± 0.3  (n=558) | 1.2 ± 0.3  (n=564) | 1.1 ± 0.4  (n=563) | 0.9 ± 0.4  (n=379) | 1.2 ± 0.5  (n=40) | 2.0 ± 0.8  (n=40) | 1.9 ± 0.8  (n=40) | 1.8 ± 0.9  (n=40) | 1.5 ± 0.7  (n=28) |
| SA-AKI  Stage 2 | 1.1 ± 0.5  (n=689) | 1.8 ± 0.5  (n=673) | 1.6 ± 0.5  (n=682) | 1.4 ± 0.6  (n=680) | 1.3 ± 0.6  (n=457) | 1.2 ± 0.5  (n=32) | 1.9 ± 0.7  (n=32) | 1.7 ± 0.6  (n=32) | 1.6 ± 0.7  (n=32) | 1.3 ± 0.6  (n=23) |
| SA-AKI  Stage 3 | 1.3 ± 0.8  (n=981) | 2.9 ± 1.9  (n=967) | 2.2 ± 1.3  (n=955) | 1.7 ± 1.0  (n=932) | 1.7 ± 1.4  (n=753) | 1.7 ± 1.2  (n=259) | 4.5 ± 2.2  (n=253) | 3.1 ± 1.6  (n=255) | 2.1 ± 1.3  (n=254) | 1.9 ± 1.3  (n=194) |

e-Figure 3. Trajectory of AKI severity in patients with SA-AKI


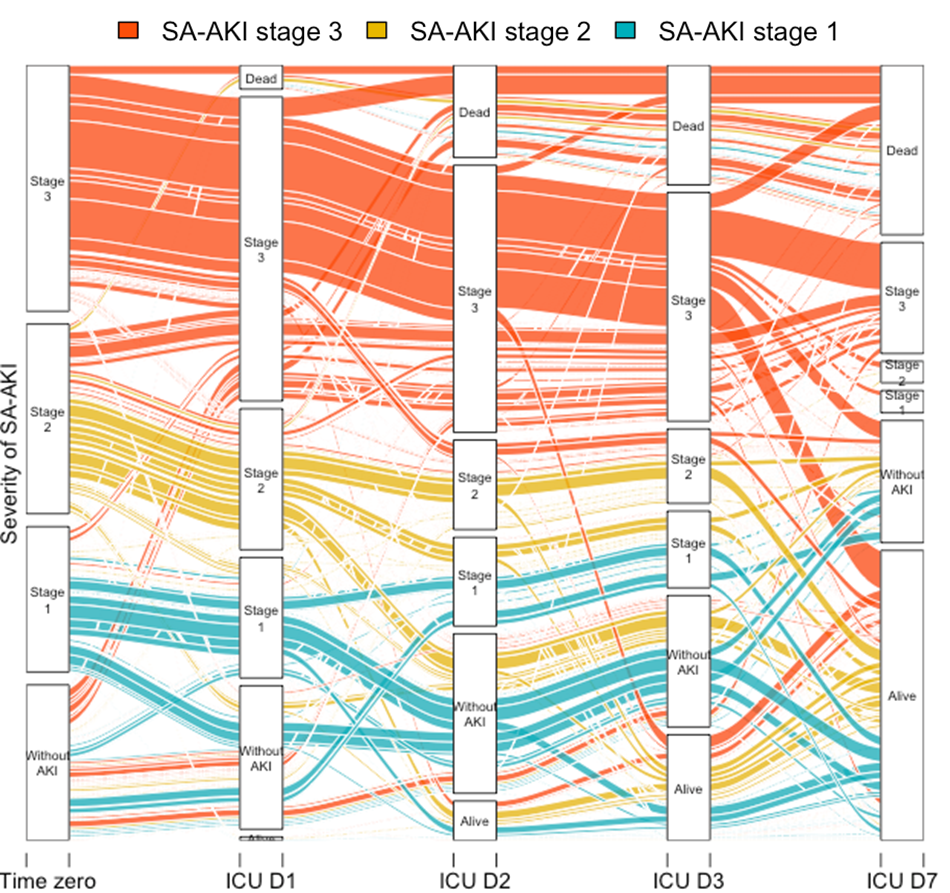


e-Figure 4. Risk difference for in-hospital mortality and ICU mortality according to the severity of SA-AKI relative to those without SA-AKI

(A) In-hospital mortality


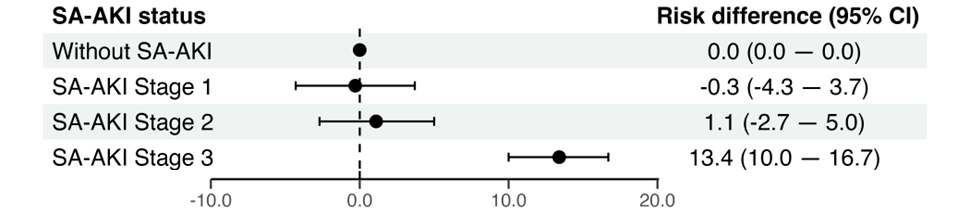


(B) ICU mortality


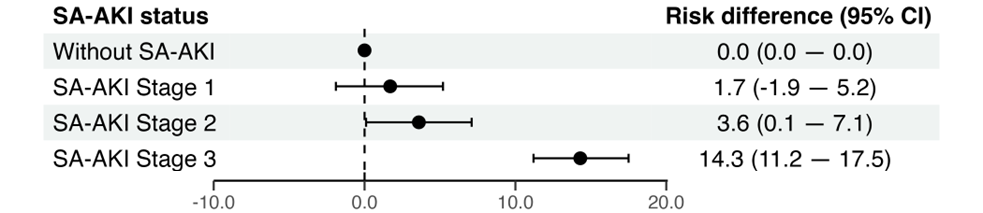


Variables included in models are those included in multivariable logistic regression for in-hospital mortality (Table 3)

CI, confidence interval; ICU, intensive care unit; SA-AKI, sepsis-associated acute kidney injury

e-Figure 5. Estimated odds ratio for in-hospital mortality based on the cumulative dose of fluid intake from time zero to ICU day 1

(A) Estimated odds ratio for in-hospital mortality in the total population


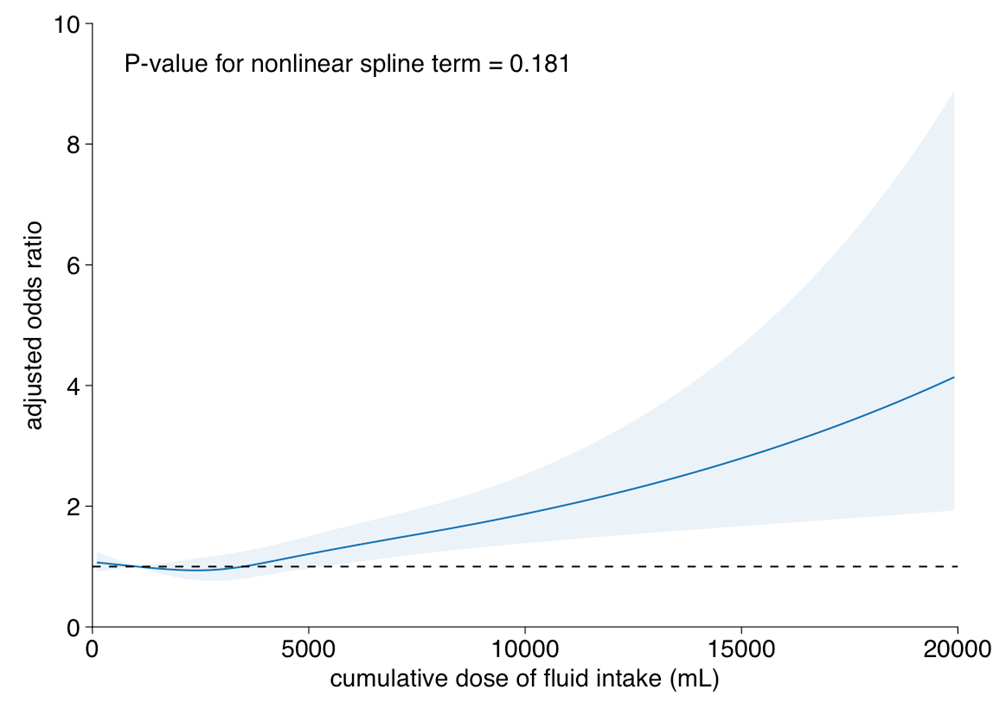


(B) Estimated odds ratio for in-hospital mortality in severe SA-AKI


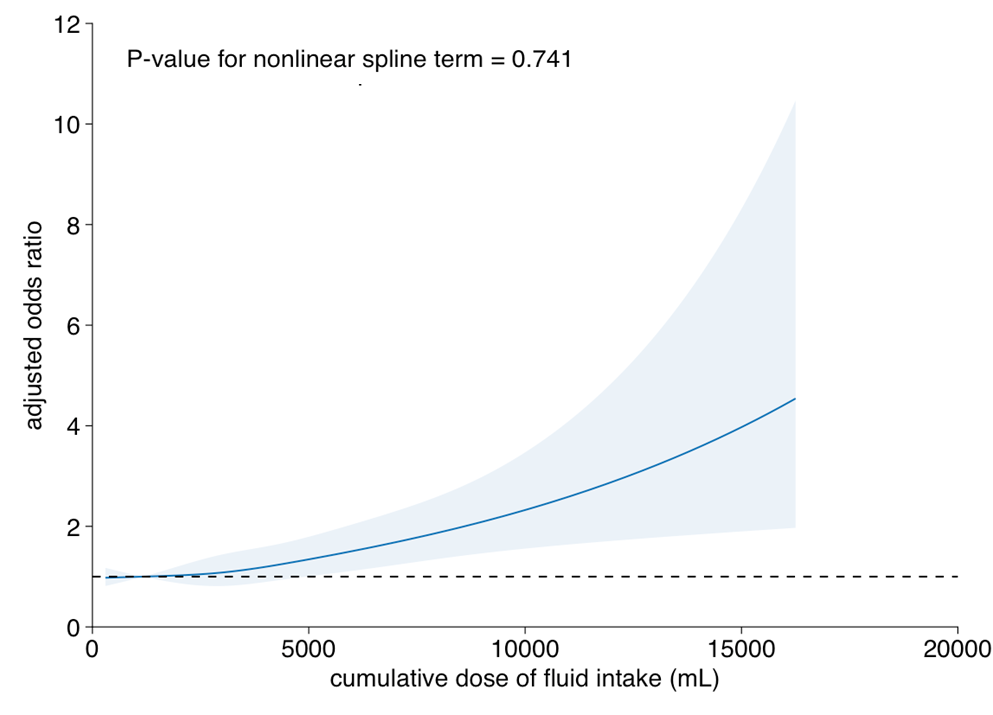


Variables included in models are those included in multivariable logistic regression for in-hospital mortality (Table 3) and cumulative output from time zero to ICU day 1.

SA-AKI, sepsis-associated acute kidney injury

e-Figure 6. Estimated odds ratio for in-hospital mortality based on the cumulative dose of fluid intake from time zero to ICU day 3

(A) Estimated odds ratio for in-hospital mortality in the total population


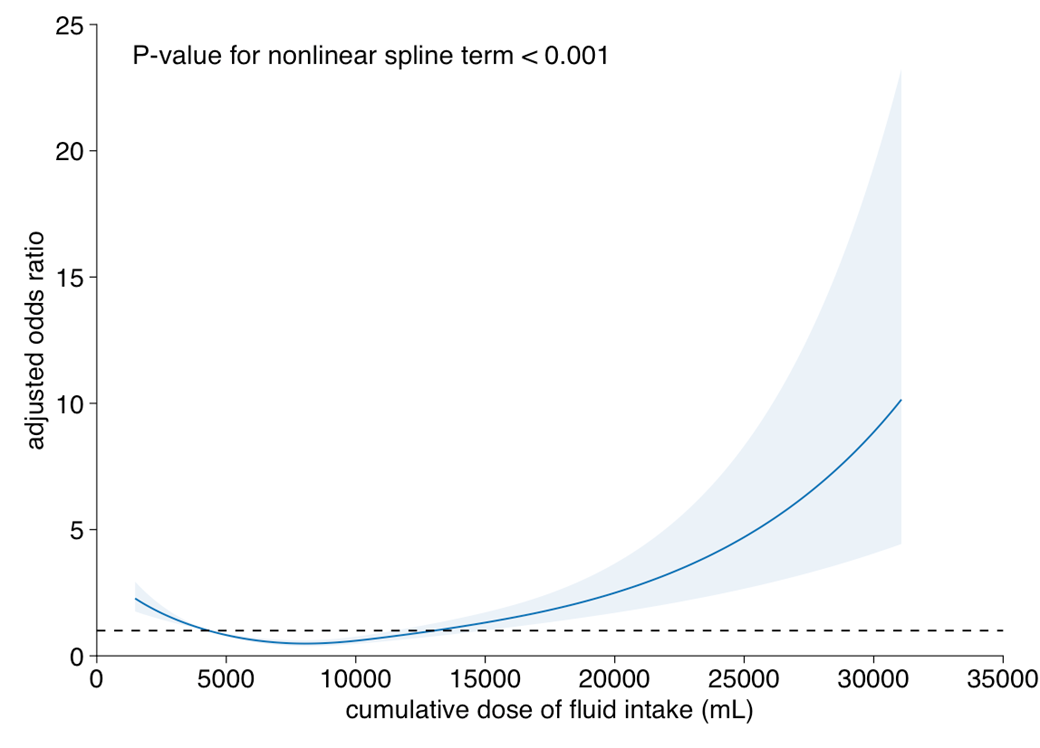


(B) Estimated odds ratio for in-hospital mortality in severe SA-AKI


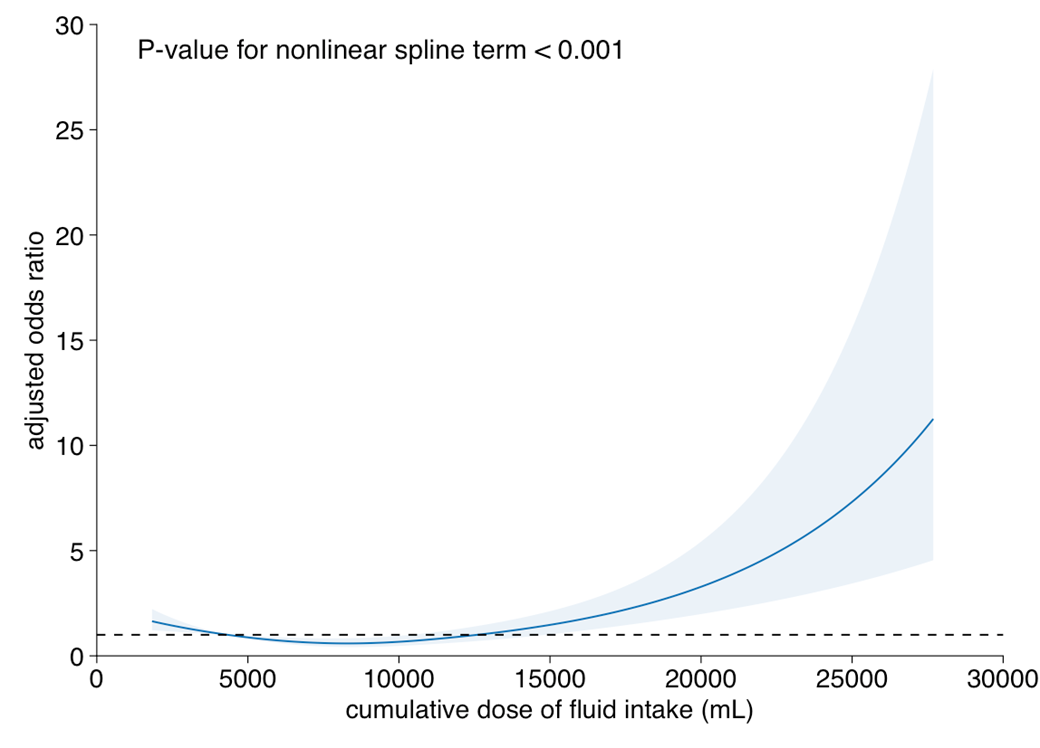


Variables included in models are those included in multivariable logistic regression for in-hospital mortality (Table 3) and cumulative output from time zero to ICU day 3

SA-AKI, sepsis-associated acute kidney injury

e-Table 1. Missing values

| Variable | N | rate(%) |
| --- | --- | --- |
| Procalcitonin at time zero | 1273 | 25.0 |
| 1Hr bundle_Blood cultures | 1061 | 20.8 |
| C-reactive protein at time zero | 449 | 8.8 |
| Input on ICU day 3 | 419 | 8.2 |
| Output on ICU day 3 | 417 | 8.2 |
| Creatinine level_last day of ICU | 144 | 6.7 |
| Pre-ICU Output | 253 | 5.0 |
| Pre-ICU Input | 181 | 3.5 |
| eGFR at ICU admission | 114 | 2.3 |
| Creatinine level_ICU day 1 | 114 | 2.3 |
| Body mass index | 111 | 2.2 |
| Lactate at time zero | 61 | 1.2 |
| Septic shock on ICU day 1 | 55 | 1.1 |
| Input on ICU day 1 | 51 | 1.0 |
| Input on ICU day 2 | 44 | 0.9 |
| Output on ICU day 2 | 44 | 0.9 |
| Appropriateness of empirical antibiotics | 42 | 0.8 |
| Output on ICU day 1 | 31 | 0.6 |
| Creatinine level_ICU day 2 | 13 | 0.3 |
| eGFR at time zero | 13 | 0.3 |
| Creatinine level_time zero | 13 | 0.3 |
| Mean blood pressure at time zero | 4 | 0.1 |
| Heart rate at time zero | 3 | 0.1 |
| Temperature at time zero | 4 | 0.1 |
| 1Hr bundle_Broad spectrum antibiotics | 2 | 0.0 |
| SAPS 3 on ICU day 1 | 1 | 0.0 |
| Duration of invasive ventilation | 1 | 0.0 |
| Creatinine level_ICU day 3 | 10 | 0.0 |
| ICU length of stay | 0 | 0.0 |
| Hospital length of stay | 0 | 0.0 |
| Age, year | 0 | 0.0 |
| Sex | 0 | 0.0 |
| Diabetes mellitus | 0 | 0.0 |
| Cardiovascular disease | 0 | 0.0 |
| Chronic lung disease | 0 | 0.0 |
| Chronic kidney disease | 0 | 0.0 |
| Solid malignant tumors | 0 | 0.0 |
| Hematological malignancies | 0 | 0.0 |
| Immunocompromised | 0 | 0.0 |
| Charlson Comorbidity Index | 0 | 0.0 |
| Clinical Frailty Score | 0 | 0.0 |
| Type Of Infection | 0 | 0.0 |
| Surgical admission among hospital onset sepsis | 0 | 0.0 |
| Site of infection_Respiratory | 0 | 0.0 |
| Site of infection_Abdominal | 0 | 0.0 |
| Site of infection_Urinary tract | 0 | 0.0 |
| Site of infection_Skin and soft tissue | 0 | 0.0 |
| Site of infection_Others | 0 | 0.0 |
| Bacterial blood sepsis | 0 | 0.0 |
| Multidrug-resistant bacteria | 0 | 0.0 |
| SOFA score at time zero | 0 | 0.0 |
| Non-renal SOFA score at time zero | 0 | 0.0 |
| SOFA score on ICU day 1 | 0 | 0.0 |
| Invasive ventilation on ICU D1 | 0 | 0.0 |
| Time from hospital admission to ICU admission | 0 | 0.0 |
| Time from diagnosis of sepsis to ICU admission | 0 | 0.0 |
| In-hospital mortality | 0 | 0.0 |
| ICU mortality | 0 | 0.0 |
| RRT at ICU discharge | 0 | 0.0 |
| Invasive ventilation during ICU stay | 0 | 0.0 |
| CRRT during ICU stay | 0 | 0.0 |
| Antibiotics before time zero | 0 | 0.0 |
| Use of nephrotoxic antibiotics | 0 | 0.0 |
| Adjunctive steroid | 0 | 0.0 |
| Source control | 0 | 0.0 |
| Vasopressor support (ICUD1) | 0 | 0.0 |
| Inotrope support (ICUD1) | 0 | 0.0 |
| Vasopressor support (during ICU stay) | 0 | 0.0 |
| Inotrope support (during ICU stay) | 0 | 0.0 |
| 1Hr bundle_Lactate measurement | 0 | 0.0 |
| 1Hr bundle_Fluid resuscitation | 0 | 0.0 |
| 1Hr bundle_Vasopressors | 0 | 0.0 |
| Creatinine level_ICU day 7 | 0 | 0.0 |

CRRT, continuous replacement therapy; eGFR, estimated glomerular filtration rate; RRT, renal replacement therapy; SAPS, Simplified Acute Physiology Score; SOFA, Sequential Organ Failure Assessment; ICU, intensive care unit

e-Table 2. Baseline characteristics of patients admitted to the general ward, categorized by their AKI status at time zero

| Variables | | | Patients in general ward  (n=6,059) | With SA-AKI (n=2,047) | Without SA-AKI (n=4,012) | *P* value |
| --- | --- | --- | --- | --- | --- | --- |
| Age, year | | | 71.9 ± 14.0 | 74.4 ± 12.8 | 70.6 ± 14.4 | <0.001 |
| Sex | |  |  |  |  | <0.001 |
|  | Male | | 3,366 (55.6) | 1,053 (51.4) | 2,313 (57.7) |  |
|  | Female | | 2,693 (44.4) | 994 (48.6) | 1,699 (42.3) |  |
| Body mass index (kg/m²) | | | 21.5 ± 4.1 | 21.9 ± 4.0 | 21.3 ± 4.0 | <0.001 |
| Comorbidities | | |  |  |  |  |
|  | Diabetes mellitus | | 1,925 (31.8) | 737 (36.0) | 1,188 (29.6) | <0.001 |
|  | Cardiovascular disease | | 1,061 (17.5) | 424 (20.7) | 637 (15.9) | <0.001 |
|  | Chronic lung disease | | 763 (12.6) | 209 (10.2) | 554 (13.8) | <0.001 |
|  | Chronic kidney disease | | 469 (7.7) | 122 (6.0) | 347 (8.7) | <0.001 |
|  | Solid malignant tumors | | 2,547 (42.0) | 837 (40.9) | 1,710 (42.6) | 0.206 |
|  | Hematological malignancies | | 452 (7.5) | 119 (5.8) | 333 (8.3) | <0.001 |
|  | Immunocompromised | | 194 (3.2) | 44 (2.1) | 150 (3.7) | 0.001 |
|  | Charlson Comorbidity Index | | 5.9 ± 2.5 | 6.0 ± 2.4 | 5.8 ± 2.6 | 0.052 |
| Clinical Frailty Score | | |  |  |  | <0.001 |
|  | 1~3 | | 1,436 (23.7) | 416 (20.3) | 1,020 (25.4) |  |
|  | 4~6 | | 2,029 (33.5) | 786 (38.4) | 1,243 (31.0) |  |
|  | 7~9 | | 2,594 (42.8) | 845 (41.3) | 1,749 (43.6) |  |
| **Characteristics of infection** | | |  |  |  |  |
| Type of infection | | |  |  |  | <0.001 |
|  | Community-onset sepsis | | 3,512 (58.0) | 1,307 (63.8) | 2,205 (55.0) |  |
|  | Hospital-onset sepsis | | 2,547 (42.0) | 740 (36.2) | 1,807 (45.0) |  |
|  |  | Surgical admission | 201 (3.3) | 63 (3.1) | 138 (3.4) | 0.504 |
| Site of infection^*^ | | |  |  |  |  |
|  | Respiratory | | 2,603 (43.0) | 760 (37.1) | 1,843 (45.9) | <0.001 |
|  | Abdominal | | 1,771 (29.2) | 598 (29.2) | 1,173 (29.2) | >0.999 |
|  | Urinary tract | | 1,283 (21.2) | 574 (28.0) | 709 (17.7) | <0.001 |
|  | Skin and soft tissue | | 159 (2.6) | 56 (2.7) | 103 (2.6) | 0.762 |
|  | Others | | 80 (1.3) | 15 (0.7) | 65 (1.6) | 0.006 |
|  | Unknown | | 637 (10.5) | 232 (11.3) | 405 (10.1) | 0.149 |
| Bacteremia | | | 2,561 (42.3) | 955 (46.7) | 1,606 (40.0) | <0.001 |
|  | Gram positive bacteremia | | 691 (11.4) | 238 (11.6) | 453 (11.3) | 0.729 |
|  | Gram negative bacteremia | | 2,138 (35.3) | 810 (39.6) | 1,328 (33.1) | <0.001 |
| Multidrug-resistant bacteria^†^ | | | 782 (12.9) | 285 (13.9) | 497 (12.4) | 0.1 |
| **Characteristics at diagnosis of sepsis** | | | |  |  |  |
| Vital signs | | |  |  |  |  |
|  | Mean blood pressure (mmHg) | | 71.7 ± 19.8 | 68.2 ± 19.4 | 73.5 ± 19.7 | <0.001 |
|  | Heart rate (/min) | | 106.5 ± 24.9 | 103.5 ± 25.5 | 108.0 ± 24.5 | <0.001 |
|  | Temperature (°C) | | 37.4 ± 1.3 | 37.2 ± 1.3 | 37.6 ± 1.2 | <0.001 |
| Septic shock | | | 651 (10.7) | 258 (12.6) | 393 (9.8) | <0.001 |
| SOFA score | | | 5.8 ± 2.6 | 7.0 ± 2.8 | 5.2 ± 2.3 | <0.001 |
| Renal SOFA score | | | 0.8 ± 1.0 | 1.7 ± 0.9 | 0.3 ± 0.6 | <0.001 |
| Non-renal SOFA score | | | 5.0 ± 2.3 | 5.3 ± 2.6 | 4.9 ± 2.2 | <0.001 |
| Outcomes of patients | | |  |  |  |  |
|  | In-hospital mortality | | 1,643 (27.1) | 659 (32.2) | 984 (24.5) | <0.001 |
|  | Time zero to discharge (days) | | 10.0 (6.0—17.0) | 10.0 (5.0—16.0) | 10.0 (6.0—18.0) | <0.001 |

Data are presented as n (%), mean ± standard deviation, or median (interquartile range)

^*^Mutually nonexclusive

^†^Detailed explanation in Supplemental Method 2

SA-AKI, sepsis-associated acute kidney injury; SOFA, Sequential Organ Failure Assessment;

e-Table 3. Baseline characteristics of the study population by SA-AKI stage

| Variables | | | All patients  (n=5,100) | Without SA-AKI (n=1,923) | SA-AKI Stage 1 (n=613) | SA-AKI Stage 2 (n=721) | SA-AKI Stage 3 (n=1,843) | *P* value |
| --- | --- | --- | --- | --- | --- | --- | --- | --- |
| Age, year | |  | 71.2 ± 13.8 | 69.6 ± 14.4 | 73.2 ± 12.6 | 74.2 ± 12.9 | 71.1 ± 13.5 | <0.001 |
| Sex, male | | | 2,960 (58.0) | 1,183 (61.5) | 363 (59.2) | 358 (49.7) | 1,056 (57.3) | <0.001 |
| Body mass index, kg/m² | | | 22.6 ± 30.5 | 21.2 ± 4.4 | 25.6 ± 87.0 | 22.5 ± 4.2 | 23.0 ± 4.7 | <0.001 |
| Comorbidities | | |  |  |  |  |  |  |
|  | Diabetes mellitus | | 1,834 (36.0) | 573 (29.8) | 224 (36.5) | 265 (36.8) | 772 (41.9) | <0.001 |
|  | Cardiovascular disease | | 957 (18.8) | 296 (15.4) | 112 (18.3) | 163 (22.6) | 386 (20.9) | <0.001 |
|  | Chronic lung disease | | 535 (10.5) | 235 (12.2) | 58 (9.5) | 80 (11.1) | 162 (8.8) | 0.005 |
|  | Chronic kidney disease | | 522 (10.2) | 118 (6.1) | 40 (6.5) | 32 (4.4) | 332 (18.0) | <0.001 |
|  | Solid malignant tumors | | 1,480 (29.0) | 609 (31.7) | 158 (25.8) | 209 (29.0) | 504 (27.3) | 0.007 |
|  | Hematological malignancies | | 375 (7.4) | 149 (7.7) | 41 (6.7) | 43 (6.0) | 142 (7.7) | 0.36 |
|  | Immunocompromised | | 204 (4.0) | 71 (3.7) | 18 (2.9) | 27 (3.7) | 88 (4.8) | 0.152 |
|  | Charlson Comorbidity Index | | 5.3 ± 2.5 | 5.1 ± 2.5 | 5.1 ± 2.3 | 5.3 ± 2.3 | 5.5 ± 2.6 | <0.001 |
| Clinical Frailty Score | | |  |  |  |  |  | <0.001 |
|  | 1~3 | | 1,288 (25.3) | 490 (25.5) | 157 (25.6) | 181 (25.1) | 460 (25.0) |  |
|  | 4~6 | | 1,962 (38.5) | 641 (33.3) | 240 (39.2) | 307 (42.6) | 774 (42.0) |  |
|  | 7~9 | | 1,850 (36.3) | 792 (41.2) | 216 (35.2) | 233 (32.3) | 609 (33.0) |  |
| **Characteristics of infection** | | |  |  |  |  |  |  |
| Type of infection | | |  |  |  |  |  | <0.001 |
|  | Community-onset sepsis | | 1,179 (23.1) | 495 (25.7) | 124 (20.2) | 100 (13.9) | 460 (25.0) |  |
|  | Hospital-onset sepsis | | 3,921 (76.9) | 1,428 (74.3) | 489 (79.8) | 621 (86.1) | 1,383 (75.0) |  |
|  |  | Surgical admission | 395 (7.7) | 186 (9.7) | 45 (7.3) | 38 (5.3) | 126 (6.8) | <0.001 |
| Site of infection^*^ | | |  |  |  |  |  |  |
|  | Respiratory | | 2,293 (45.0) | 1,041 (54.1) | 252 (41.1) | 286 (39.7) | 714 (38.7) | <0.001 |
|  | Abdominal | | 1,433 (28.1) | 484 (25.2) | 187 (30.5) | 207 (28.7) | 555 (30.1) | 0.003 |
|  | Urinary tract | | 1,070 (21.0) | 270 (14.0) | 130 (21.2) | 197 (27.3) | 473 (25.7) | <0.001 |
|  | Skin and soft tissue | | 203 (4.0) | 73 (3.8) | 18 (2.9) | 23 (3.2) | 89 (4.8) | 0.087 |
|  | Others | | 99 (1.9) | 47 (2.4) | 19 (3.1) | 11 (1.5) | 22 (1.2) | 0.005 |
|  | Unknown | | 443 (8.7) | 138 (7.2) | 47 (7.7) | 62 (8.6) | 196 (10.6) | 0.002 |
| Bacterial blood sepsis | | | 2,379 (46.6) | 793 (41.2) | 276 (45.0) | 382 (53.0) | 928 (50.4) | <0.001 |
|  | Gram positive blood sepsis | | 709 (13.9) | 255 (13.3) | 63 (10.3) | 108 (15.0) | 283 (15.4) | 0.01 |
|  | Gram negative blood sepsis | | 1,995 (39.1) | 652 (33.9) | 242 (39.5) | 327 (45.4) | 774 (42.0) | <0.001 |
| Multidrug-resistant bacteria^†^ | | | 677 (13.3) | 237 (12.3) | 90 (14.7) | 99 (13.7) | 251 (13.6) | 0.41 |
| **Characteristics at diagnosis of sepsis** | | | |  |  |  |  |  |
| Vital signs | | |  |  |  |  |  |  |
|  | Mean blood pressure (mmHg) | | 70.0 ± 21.0 | 74.7 ± 21.8 | 67.9 ± 20.3 | 66.9 ± 19.3 | 67.0 ± 20.2 | <0.001 |
|  | Heart rate (/min) | | 107.7 ± 26.7 | 110.4 ± 25.7 | 108.7 ± 26.6 | 106.5 ± 26.8 | 105.0 ± 27.3 | <0.001 |
|  | Temperature (°C) | | 37.2 ± 1.3 | 37.4 ± 1.2 | 37.4 ± 1.3 | 37.3 ± 1.3 | 37.0 ± 1.4 | <0.001 |
| eGFR at time zero (mL/min/1.73 m^2^) | | | 54.6 ± 51.2 | 91.8 ± 62.4 | 45.9 ± 17.2 | 34.7 ± 20.6 | 26.6 ± 23.4 | <0.001 |
| SOFA score | | | 7.3 ± 3.1 | 6.0 ± 2.6 | 6.8 ± 2.7 | 7.2 ± 2.7 | 8.9 ± 3.2 | <0.001 |
| Renal SOFA score | | | 1.2 ± 1.2 | 0.2 ± 0.5 | 0.8 ± 0.5 | 1.4 ± 0.6 | 2.1 ± 1.2 | <0.001 |
| Non-renal SOFA score | | | 6.2 ± 2.8 | 5.8 ± 2.6 | 6.0 ± 2.6 | 5.9 ± 2.7 | 6.7 ± 3.0 | <0.001 |
| **Characteristics during ICU stay** | | |  |  |  |  |  |  |
| SOFA score on ICU day 1 | | | 9.7 ± 3.8 | 8.0 ± 3.3 | 8.9 ± 3.0 | 9.4 ± 3.3 | 11.9 ± 3.6 | <0.001 |
| eGFR at ICU admission | | | 57.6 ± 61.6 | 99.0 ± 81.0 | 48.7 ± 18.4 | 36.1 ± 16.7 | 26.1 ± 22.3 | <0.001 |
| SAPS 3 on ICU day 1 | | | 73.9 ± 15.7 | 68.9 ± 14.7 | 70.8 ± 13.7 | 72.5 ± 14.0 | 80.7 ± 15.5 | <0.001 |
| Septic shock on ICU day 1 | | | 2,456 (48.7) | 806 (42.6) | 329 (54.4) | 362 (50.6) | 959 (52.4) | <0.001 |
| Invasive ventilation on ICU day 1 | | | 2,323 (45.5) | 809 (42.1) | 243 (39.6) | 258 (35.8) | 1,013 (55.0) | <0.001 |
| Time from diagnosis of sepsis to ICU admission (hr) | | | 7.3 (4.5—13.6) | 7.4 (4.4—13.8) | 6.9 (4.6—12.9) | 7.4 (5.0—13.9) | 7.2 (4.3—13.3) | 0.216 |
| **Treatment related variables** | | |  |  |  |  |  |  |
| Antibiotics before time zero | | | 1,611 (31.6) | 674 (35.0) | 181 (29.5) | 162 (22.5) | 594 (32.2) | <0.001 |
| Appropriateness of empirical antibiotics^†^ | | | 4,499 (88.9) | 1,705 (89.3) | 535 (88.3) | 635 (89.1) | 1,624 (88.8) | 0.914 |
| Use of nephrotoxic antibiotics^†^ | | | 1,075 (21.1) | 420 (21.8) | 110 (17.9) | 112 (15.5) | 433 (23.5) | <0.001 |
| Adjunctive steroid | | | 1,139 (22.3) | 387 (20.1) | 117 (19.1) | 132 (18.3) | 503 (27.3) | <0.001 |
| Source control | | |  |  |  |  |  |  |
|  | Non-surgical source control | | 583 (11.4) | 236 (12.3) | 88 (14.4) | 86 (11.9) | 173 (9.4) | 0.003 |
|  | Surgical source control | | 263 (5.2) | 94 (4.9) | 29 (4.7) | 45 (6.2) | 95 (5.2) | 0.523 |
| Vasopressor support (ICUD1) | | | 4,044 (79.3) | 1,386 (72.1) | 496 (80.9) | 575 (79.8) | 1,587 (86.1) | <0.001 |
| Vasopressor support (during ICU stay) | | | 4,302 (84.4) | 1,495 (77.7) | 524 (85.5) | 602 (83.5) | 1,681 (91.2) | <0.001 |
| 1Hr bundle compliance, % | | |  |  |  |  |  |  |
|  | Lactate measurement | | 4,296 (84.2) | 1,607 (83.6) | 511 (83.4) | 615 (85.3) | 1,563 (84.8) | 0.559 |
|  | Blood cultures | | 2,580 (63.9) | 1,067 (67.2) | 320 (64.4) | 367 (65.3) | 826 (59.3) | <0.001 |
|  | Broad spectrum antibiotics | | 1,432 (28.1) | 599 (31.2) | 164 (26.8) | 175 (24.3) | 494 (26.8) | 0.001 |
|  | Fluid resuscitation | | 4,273 (83.8) | 1,644 (85.5) | 524 (85.5) | 620 (86.0) | 1,485 (80.6) | <0.001 |
|  | Vasopressors | | 3,188 (62.5) | 1,304 (67.8) | 393 (64.1) | 414 (57.4) | 1,077 (58.4) | <0.001 |

Data are presented as n (%), mean ± standard deviation, or median (interquartile range).

^*^Mutually nonexclusive

^†^Detailed explanation in Supplemental Method 2

eGFR, estimated glomerular filtration rate; ICU, intensive care unit; SA-AKI, sepsis-associated acute kidney injury; SAPS, Simplified Acute Physiology Score; SOFA, Sequential Organ Failure Assessment

e-Table 4. Timing of SA-AKI diagnosis

| Variables | | All SA-AKI  (n=3,177) | SA-AKI stage 1  (n=613) | SA-AKI stage 2  (n=721) | SA-AKI stage 3  (n=1,843) |
| --- | --- | --- | --- | --- | --- |
| Time from diagnosis of sepsis to any stage of SA-AKI (days) | | 0 [0–0] | 0 [0–1] | 0 [0–0] | 0 [0–0] |
| Time from diagnosis of sepsis to max stage of SA-AKI (days) | | 0 [0–1] | 0 [0–1] | 0 [0–1] | 0 [0–1] |
| Time of any stage of SA-AKI occurrence | | |  |  |  |
|  | Time zero | 2506 (78.9) | 415 (67.7) | 604 (83.8) | 1487 (80.7) |
|  | ICU day 1 | 322 (10.1) | 80 (13.1) | 40 (5.5) | 202 (11) |
|  | ICU day 2 | 246 (7.7) | 83 (13.5) | 57 (7.9) | 106 (5.8) |
|  | ICU day 3 | 65 (2.0) | 28 (4.6) | 9 (1.2) | 28 (1.5) |
|  | ICU day 7 | 38 (1.2) | 7 (1.1) | 11 (1.5) | 20 (1.1) |
| Time of max stage of SA-AKI occurrence | |  |  |  |  |
|  | Time zero | 2008 (63.2) | 415 (67.7) | 532 (73.8) | 1061 (57.6) |
|  | ICU day 1 | 563 (17.7) | 80 (13.1) | 59 (8.2) | 424 (23.0) |
|  | ICU day 2 | 381 (12.0) | 83 (13.5) | 75 (10.4) | 223 (12.1) |
|  | ICU day 3 | 145 (4.6) | 28 (4.6) | 38 (5.3) | 79 (4.3) |
|  | ICU day 7 | 80 (2.5) | 7 (1.1) | 17 (2.4) | 56 (3.0) |
| CRRT timing (in 1,225 patients diagnosed with stage 3 by RRT criteria) | | | |  | n=1225 |
|  | ICU day 1 |  |  |  | 838 (68.4) |
|  | ICU day 2 |  |  |  | 263 (21.5) |
|  | ICU day 3 |  |  |  | 71 (5.8) |
|  | ICU day 7 |  |  |  | 53 (4.3) |

Data are n (%), median (interquartile range)

CRRT, continuous renal replacement therapy; ICU, intensive care unit; SA-AKI, sepsis-associated acute kidney injury

e-Table 5. The persistence of AKI and renal function on the last day of ICU

| Variables | | All SA-AKI  (n=3,177) | SA-AKI stage 1  (n=613) | SA-AKI stage 2  (n=721) | SA-AKI stage 3  (n=1,843) |
| --- | --- | --- | --- | --- | --- |
| ^*^Transient vs persistent AKI | |  |  |  |  |
|  | Persistent | 1,715 (54.0) | 108 (17.6) | 292 (40.5) | 1,315 (71.4) |
|  | Transient | 1,289 (40.6) | 479 (78.1) | 399 (55.3) | 411 (22.3) |
|  | ^†^Not applicable | 173 (5.4) | 26 (4.2) | 30 (4.2) | 117 (8.9) |
| ^‡^Status on the last day of ICU | |  |  |  |  |
|  | Recovery | 1547 (48.7) | 520 (84.8) | 470 (65.2) | 557 (30.2) |
|  | AKD stage 1 | 284 (8.9) | 69 (11.3) | 95 (13.2) | 120 (6.5) |
|  | AKD stage 2 | 324 (10.2) | 7 (1.1) | 119 (16.5) | 198 (10.7) |
|  | AKD stage 3 | 1022 (32.2) | 17 (2.8) | 37 (5.1) | 968 (52.5) |

^*^ Transient vs. persistent AKI were defined by the Acute Disease Quality Initiative (ADQI) 16 Workgroup based on whether AKI persisted for more than 48 hours [2]. Due to limitations in our database, we were unable to apply the 48-hour criteria to define transient or persistent AKI, so the following definitions were used: Patients diagnosed with AKI on time zero or ICU day 1 were classified as transient if AKI resolved by ICU day 3, while those diagnosed on ICU days 2 or 3 were considered transient if recovery occurred by day 7.

^†^Patients who lacked follow-up creatinine measurements to confirm recovery, were classified as "not applicable".

^‡^Since AKI describes a sudden loss of kidney function limited to a duration of 7 days, we designated the renal function status on the last day of ICU stay as AKD, which is a broader term that includes conditions lasting up to 3 months.

e-Table 6. Outcomes categorized by the severity of SA-AKI

| Variables | All patients (n=5,100) | Without SA-AKI (n=1,923) | SA-AKI stage 1 (n=613) | SA-AKI stage 2 (n=721) | SA-AKI stage 3 (n=1,843) | *P* value |
| --- | --- | --- | --- | --- | --- | --- |
| In-hospital mortality | 1,843 (36.1) | 480 (25.0) | 164 (26.8) | 213 (29.5) | 986 (53.5) | <0.001 |
| ICU mortality | 1,339 (26.3) | 277 (14.4) | 109 (17.8) | 149 (20.7) | 804 (43.6) | <0.001 |
| ICU length of stay (day) | 4.5 (2.0—10.0) | 4.4 (2.0—10.2) | 4.2 (2.1—8.8) | 3.8 (1.9—7.9) | 5.1 (2.0—11.0) | 0.004 |
| Hospital length of stay (day) | 15.9 (8.2—30.6) | 17.9 (9.7—33.8) | 15.6 (8.8—28.8) | 14.0 (8.4—25.6) | 15.4 (5.6—29.8) | <0.001 |
| RRT at ICU discharge (HD or PD) | 212 (4.2) | 6 (0.3) | 2 (0.3) | 2 (0.3) | 202 (11.0) | <0.001 |
| Invasive ventilation during ICU stay (day) | 2,674 (52.4) | 912 (47.4) | 271 (44.2) | 300 (41.6) | 1,191 (64.6) | <0.001 |
| Duration of invasive ventilation (day) | 5.0 (2.0—12.0) | 6.0 (3.0—14.0) | 5.0 (2.0—13.0) | 5.0 (2.0—14.0) | 4.0 (2.0—10.0) | <0.001 |
| CRRT during ICU stay | 1,311 (25.7) | 38 (2.0) | 10 (1.6) | 23 (3.2) | 1,240 (67.3) | <0.001 |

Data are presented as n (%), mean ± standard deviation, or median (interquartile range)

CRRT, continuous renal replacement therapy; HD, hemodialysis; ICU, intensive care unit; PD peritoneal dialysis; RRT, renal replacement therapy.

e-Table 7. Results of bivariate and multivariable logistic regression analyses for in-hospital mortality in patients without SA-AKI and SA-AKI stage 1

| Variables | | Bivariate analysis | | |  | Multivariable Logistic Regression Analysis^*,†^ | |
| --- | --- | --- | --- | --- | --- | --- | --- |
|  |  | Survival (n=1,892) | Non-survival (n= 644) | *P* value |  | Adjusted odds ratio (95% CI) | *P* value |
| Age | | 70.1 ± 14.2 | 71.8 ± 13.8 | 0.007 |  | 1.00 (1.0-1.01) | 0.471 |
| Sex, male | | 1,137 (60.1) | 409 (63.5) | 0.137 |  | 0.90 (0.73-1.11) | 0.347 |
| Body mass index | | 21.6 ± 4.4 | 24.2 ± 85.3 | <0.001 |  |  |  |
| Clinical Frailty Score | |  |  | <0.001 |  |  |  |
|  | 1~3 | 529 (28.0) | 118 (18.3) |  |  |  |  |
|  | 4~6 | 654 (34.6) | 227 (35.2) |  |  |  |  |
|  | 7~9 | 709 (37.5) | 299 (46.4) |  |  |  |  |
| Comorbidity | |  |  |  |  |  |  |
|  | Diabetes mellitus | 606 (32.0) | 191 (29.7) | 0.284 |  |  |  |
|  | Cardiovascular disease | 292 (15.4) | 116 (18.0) | 0.14 |  |  |  |
|  | Chronic lung disease | 213 (11.3) | 80 (12.4) | 0.467 |  |  |  |
|  | Chronic kidney disease | 123 (6.5) | 35 (5.4) | 0.383 |  |  |  |
|  | Solid malignant tumors | 534 (28.2) | 233 (36.2) | <0.001 |  |  |  |
|  | Hematological malignancies | 109 (5.8) | 81 (12.6) | <0.001 |  | 1.72 (1.20-2.47) | 0.003 |
|  | Immunocompromised | 59 (3.1) | 30 (4.7) | 0.087 |  |  |  |
| SOFA score on ICU day 1 | | 7.8 ± 3.1 | 9.6 ± 3.3 | <0.001 |  | 1.07 (1.03-1.12) | 0.001 |
| SAPS3 | | 66.8 ± 13.3 | 77.0 ± 15.2 | <0.001 |  | 1.04 (1.03-1.05) | <0.001 |
| Septic shock on ICU day 1 | | 825 (44.3) | 310 (48.6) | 0.069 |  |  |  |
| Type of infection | |  |  | 0.647 |  |  |  |
|  | Hospital-onset sepsis | 457 (24.2) | 162 (25.2) |  |  |  |  |
|  | Community-onset sepsis | 1,435 (75.8) | 482 (74.8) |  |  |  |  |
| Site of infection^‡^ | |  |  |  |  |  |  |
|  | Respiratory | 858 (45.3) | 435 (67.5) | <0.001 |  | 2.09 (1.67-2.60) | <0.001 |
|  | Abdominal | 551 (29.1) | 120 (18.6) | <0.001 |  |  |  |
|  | Urinary tract | 348 (18.4) | 52 (8.1) | <0.001 |  |  |  |
| Bacterial blood sepsis | |  |  |  |  |  |  |
|  | Gram positive blood sepsis | 238 (12.6) | 80 (12.4) | 0.972 |  |  |  |
|  | Gram negative blood sepsis | 701 (37.1) | 193 (30.0) | 0.001 |  |  |  |
| Multidrug-resistant bacteria^§^ | | 239 (12.6) | 88 (13.7) | 0.544 |  |  |  |
| Antibiotics before time zero | | 631 (33.4) | 224 (34.8) | 0.538 |  |  |  |
| Appropriateness of empirical antibiotics^§^ | | 1,694 (90.1) | 546 (85.8) | 0.004 |  |  |  |
| 1Hr bundle compliance, % | |  |  |  |  |  |  |
|  | Broad spectrum antibiotics | 565 (29.9) | 198 (30.8) | 0.693 |  |  |  |
|  | Fluid resuscitation | 1,628 (86.0) | 540 (83.9) | 0.193 |  |  |  |
|  | Vasopressors | 1,280 (67.7) | 417 (64.8) | 0.192 |  |  |  |
| Use of nephrotoxic antibiotics^§^ | | 372 (19.7) | 158 (24.5) | 0.01 |  |  |  |
| Adjunctive steroid | | 326 (17.2) | 178 (27.6) | <0.001 |  | 1.29 (1.02-1.65) | 0.036 |
| Source control (either surgical or non-surgical) | | 378 (20.0) | 69 (10.7) | <0.001 |  | 0.73 (0.54-0.99) | 0.044 |
| Vasopressors on ICU day 1 | | 1,387 (73.3) | 495 (76.9) | 0.084 |  |  |  |
| Invasive mechanical ventilation on ICU day 1 | | 687 (36.3) | 365 (56.7) | <0.001 |  |  |  |

Data are presented as n (%) or mean ± standard deviation

^*^Variables included the multivariable model were selected through the least absolute shrinkage and selection operator (LASSO) regression for variables in the bivariate analysis.

^†^Multilevel logistic regression model was adopted to adjust variability between hospitals

^‡^ Mutually nonexclusive

^§^Detailed explanation in Supplemental Method 2

CI, confidence interval; ICU, intensive care unit; SA-AKI, sepsis-associated acute kidney injury

e-Table 8. Sensitivity analysis: SA-AKI incidence and impact of one-hour fluid resuscitation using three different methods for assuming the baseline creatinine value

| Variables | | Main result | Sensitivity analysis 1^*^ | Sensitivity analysis 2^†^ | Sensitivity analysis 3^‡^ | Sensivity analysis 4^§^ | Sensivity analysis 5^ll^ | Sensitivity analysis 6^**^ |
| --- | --- | --- | --- | --- | --- | --- | --- | --- |
| SA-AKI incidence | |  |  |  |  |  |  |  |
|  | SA-AKI stage 1 | 613 (12.0) | 577 (11.3) | 569 (11.2) | 604 (11.8) | 619 (12.1) | 617 (12.1) | 612 (12.0) |
|  | SA-AKI stage 2 | 721 (14.1) | 639 (12.5) | 691 (13.5) | 756 (14.8) | 758 (14.9) | 735 (14.4) | 710 (13.9) |
|  | SA-AKI stage 3 | 1843 (36.1) | 1685 (33.0) | 1761 (34.5) | 1902 (37.3) | 1849 (36.3) | 1837 (36.0) | 1836 (36.0) |
|  | Any stage of SA-AKI | 3177 (62.3) | 2901 (56.9) | 3021 (59.2) | 3262 (64.0) | 3226 (63.3) | 3189 (62.5) | 3158 (61.9) |
| Adjusted odds ratio (95% CI) for in‑hospital mortality associated with one-hour fluid resuscitation adherence^††, ***^ | | | | | | | | |
|  | Overall | 0.72 (0.61-0.86) | 0.72 (0.61-0.86) | 0.72 (0.61-0.86) | 0.72 (0.61-0.86) | 0.72 (0.61-0.86) | 0.72 (0.61-0.86) | 0.72 (0.61-0.86) |
|  | Severe SA-AKI | 0.62 (0.48-0.79) | 0.60 (0.47-0.77) | 0.58 (0.46-0.75) | 0.61 (0.48-0.77) | 0.62 (0.49-0.79) | 0.58 (0.45-0.73) | 0.61 (0.48-0.78) |
|  | Without SA-AKI or SA-AKI stage 1 | 0.95 (0.72-1.25) | 0.90 (0.69-1.17) | 0.92 (0.70-1.20) | 0.95 (0.71-1.26) | 0.97 (0.73-1.28) | 0.97 (0.73-1.28) | 0.96 (0.73-1.26) |

^*^Assume the estimated GFR to be 65 mL/min/1.73 m2 to assume the baseline creatinine value in patients without CKD and the nadir creatinine for patients with CKD

^†^Assume the estimated GFR to be 70 mL/min/1.73 m2 to assume the baseline creatinine value in patients without CKD and the nadir creatinine for patients with CKD

^‡^Assume the estimated GFR to be 75 mL/min/1.73 m2 in all patients

^§^Assume the estimated GFR to be 75 mL/min/1.73 m2 in patients without CKD and the estimated GFR of 50 mL/min/1.73 m2 for patients with CKD.

^ll^Assume the estimated GFR to be 75 mL/min/1.73 m2 in patients without CKD and the estimated GFR of 40 mL/min/1.73 m2 for patients with CKD.

^**^Assume the estimated GFR to be 75 mL/min/1.73 m2 in patients without CKD and the estimated GFR of 30 mL/min/1.73 m2 for patients with CKD.

^††^The variables included in the models are those included in multivariable logistic regression for in-hospital mortality (Table 3)

^***^Multilevel logistic regression model was adopted to adjust variability between hospitals.

CKD, chronic kidney disease; CI, confidence interval; SA-AKI, sepsis-associated acute kidney injury

**References**

1. Li A, Ling L, Qin H, Arabi YM, Myatra SN, Egi M, et al. Epidemiology, Management, and Outcomes of Sepsis in ICUs among Countries of Differing National Wealth across Asia. Am J Respir Crit Care Med. 2022;206:1107-16.

2. Chawla LS, Bellomo R, Bihorac A, Goldstein SL, Siew ED, Bagshaw SM, et al. Acute kidney disease and renal recovery: consensus report of the Acute Disease Quality Initiative (ADQI) 16 Workgroup. Nat Rev Nephrol. 2017;13:241-57.
